# The circularly permuted globin domain of androglobin exhibits atypical heme stabilization and nitric oxide interaction†

**DOI:** 10.1039/d4sc00953c

**Published:** 2024-03-21

**Authors:** Brandon J. Reeder, Giuseppe Deganutti, John Ukeri, Silvia Atanasio, Dimitri A. Svistunenko, Christopher Ronchetti, Juan Carlos Mobarec, Elizabeth Welbourn, Jeffrey Asaju, Marten H. Vos, Michael T. Wilson, Christopher A. Reynolds

**Affiliations:** a School of Life Sciences, University of Essex Wivenhoe Park Colchester Essex CO4 3SQ UK reedb@essex.ac.uk; b Centre for Health and Life Sciences (CHLS) Alison Gingell Building Coventry CV1 5FB UK ad5291@coventry.ac.uk; c LOB, CNRS, INSERM, École Polytechnique, Institut Polytechnique de Paris 91128 Palaiseau France

## Abstract

In the decade since the discovery of androglobin, a multi-domain hemoglobin of metazoans associated with ciliogenesis and spermatogenesis, there has been little advance in the knowledge of the biochemical and structural properties of this unusual member of the hemoglobin superfamily. Using a method for aligning remote homologues, coupled with molecular modelling and molecular dynamics, we have identified a novel structural alignment to other hemoglobins. This has led to the first stable recombinant expression and characterization of the circularly permuted globin domain. Exceptional for eukaryotic globins is that a tyrosine takes the place of the highly conserved phenylalanine in the CD1 position, a critical point in stabilizing the heme. A disulfide bond, similar to that found in neuroglobin, forms a closed loop around the heme pocket, taking the place of androglobin's missing CD loop and further supporting the heme pocket structure. Highly unusual in the globin superfamily is that the heme iron binds nitric oxide as a five-coordinate complex similar to other heme proteins that have nitric oxide storage functions. With rapid autoxidation and high nitrite reductase activity, the globin appears to be more tailored toward nitric oxide homeostasis or buffering. The use of our multi-template profile alignment method to yield the first biochemical characterisation of the circularly permuted globin domain of androglobin expands our knowledge of the fundamental functioning of this elusive protein and provides a pathway to better define the link between the biochemical traits of androglobin with proposed physiological functions.

## Abstract 2

Since the discovery of androglobin, a multi-domain hemoglobin associated with ciliogenesis and spermatogenesis, there has been little advance in the knowledge of the biochemical and structural properties of this member of the hemoglobin superfamily.

## Graphical abstract

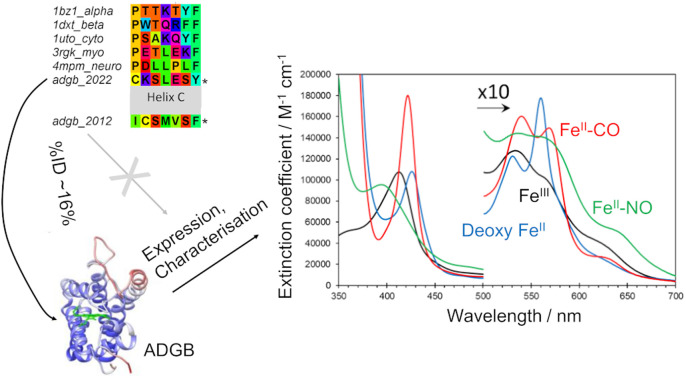

## Introduction

The hemoglobin (Hb) of the erythrocyte is one of the most studied proteins in science. However, a decade after the discovery of androglobin (Adgb), a multi-domain Hb first identified in the testis of metazoans, there is still very little known about this novel member of the Hb superfamily. Adgb is unusual due to its long length of 1667 amino acids (human), with a centrally positioned globin domain.^1^ This compares to the 140–190 amino acids of other human globins such as the erythrocyte Hb,^2^ myoglobin (Mb), neuroglobin (Ngb)^3^ and cytoglobin (Cygb)^4^ and is significantly larger than other multi-domain globins such as flavohemoglobin.^5^ Initial sequence alignment analysis predicted the heme-binding globin domain of Adgb (Adgb-GD) to consist of an eight alpha-helical structure (termed A to H) with a 3-on-3 alpha-helical fold that encloses the heme moiety and is typical of most non-truncated globin architectures.^1^ However, highly unusual in the globin family is that Adgb-GD is circularly permuted with a calmodulin binding domain situated between the H and A helices.^1^ The N-terminal region of Adgb is reported to contain a calpain-7-like region and the C-terminal region is reported to contain sequences for a coiled-coil region, a nuclear localization signal (NLS) and an ER membrane endoplasmic reticulum retention signal.^1^ Calpain C2-like domains flanking the globin domain are predicted from calpain homologues studies.^6^

The potential interaction of Adgb with nitric oxide (NO) and calcium may underlie its relevance to spermatogenesis and ciliogenesis, as well as to potential diseases. A knockdown study in cells showed enhanced apoptosis and proliferation inhibition in glioma cell lines, relating to changes in the level of several proteins involved in cell proliferation, survival or apoptosis, including STAT3 cleaved caspase-3 and Bcl-2.^7^ Additionally, STXBP5 antisense proficient GR pancreatic cancer cell lines overexpress Adgb through ADGB promotor methylation, leading to drug resistance and inhibition of cell apoptosis. Recently, mRNA-Seq data from mammalian tissue have shown that Adgb is expressed in the female reproductive tract, lungs, and brain. In each case, Adgb is specifically associated with cell types forming motile cilia, with expression linked to transcription factors involved in ciliogenesis.^8^ An absence of Adgb leads to defective sperm head and flagellum formation during spermatogenesis.^9^

There remains a lack of data concerning the structure, properties or functions of this protein. This lack of knowledge on the structural and functional aspects of the protein is due to the difficulty in generating such a large full-length hemoglobin by recombinant techniques and the instabilities of the heme-binding, circularly permuted globin domain.^10^

The alignment between Adgb and other proteins with regard to Adgb-GD was reassessed using a novel helix alignment method shown to work in the twilight zone of low sequence similarity.^11–13^ Our unique identification of the globin domain yielded a structure by homology modelling that was stable for initial 500 ns of molecular dynamics simulations, giving support for the potential expression of this protein despite initial negative expression results.^10^ Thus, based on our new alternative alignment, we have expressed the circularly permuted globin domain as a stable recombinant protein. An intramolecular disulfide bond linking the N and C terminal sections of the heme binding globin domain appears to stabilize the ‘CD loop’ heme pocket region and likely the whole globular domain.

With a stable form of the protein expressed, we have characterized the protein using optical, electron paramagnetic resonance (EPR), stopped-flow and femtosecond laser flash photolysis spectroscopy to show that Adgb binds NO as a five-coordinate heme iron. This is unlike other globins, but similar to that observed with other hemoproteins such as guanylate cyclase, cytochrome c prime (cyt c′) and dissimilatory nitrate respiration regulator.^14–16^ Furthermore, the protein exhibits high nitrite reductase activity, which is influenced by the redox state of the disulfide bond and exhibits a high autoxidation rate similar to Ngb. Based on our findings we propose that the globin domain may serve as an NO storage or NO synthesis protein under hypoxic conditions. With a binding domain for a calcium-dependent calmodulin and a calcium-dependent calpain-like sequence on the N-terminal domain, Adgb likely plays a role in both NO homeostasis and calcium signaling, with repercussions on spermatogenesis, sperm maturation and motile cilia function.^17–20^

## Results and discussion

### The alignment of the androglobin helices to known templates

**Fig. 1:**
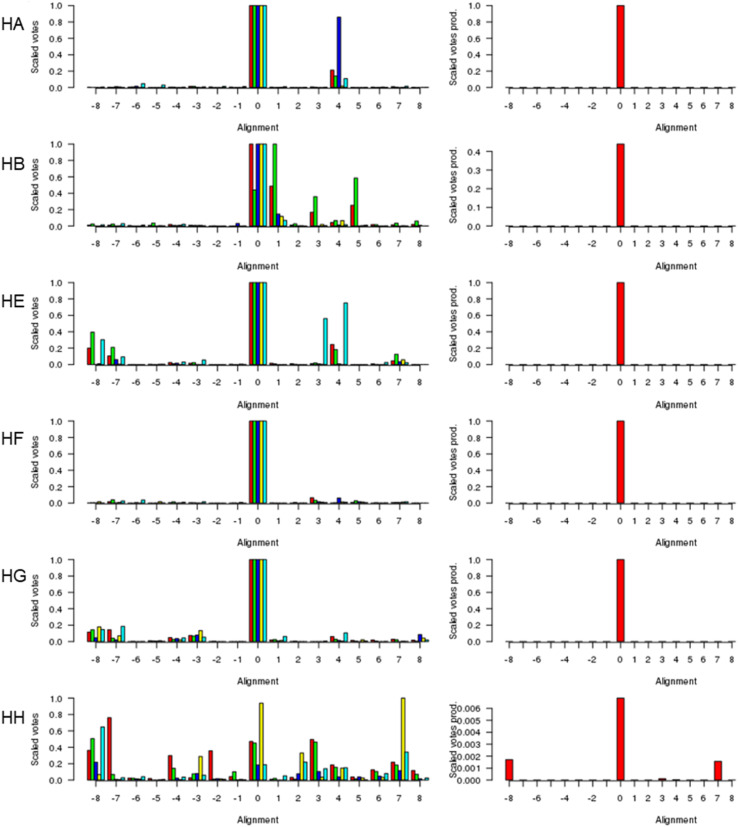
The alignment of the androglobin heme-binding globin domain helices. The left-hand column gives the individual alignments for helices A–B and helices E–H, one per row (denoted HA, HB, *etc.*). The alpha-Hb (red), beta-Hb (green), Cygb (blue), Mb (yellow), and Ngb (cyan) alignments to Adgb are denoted by the bars representing the number of votes (scaled between 0 and 1). The right-hand column gives the consensus alignment where the votes for the individual alignments are multiplied together. The results point overwhelmingly to the 0 alignment given in Fig. 2. An alignment of +1 would correspond to the movement of the Adgb helix one residue to the right in Fig. 2; an alignment of say −3 would correspond to the movement of the Adgb helix three residues to the left in Fig. 2.

**Fig. 2:**
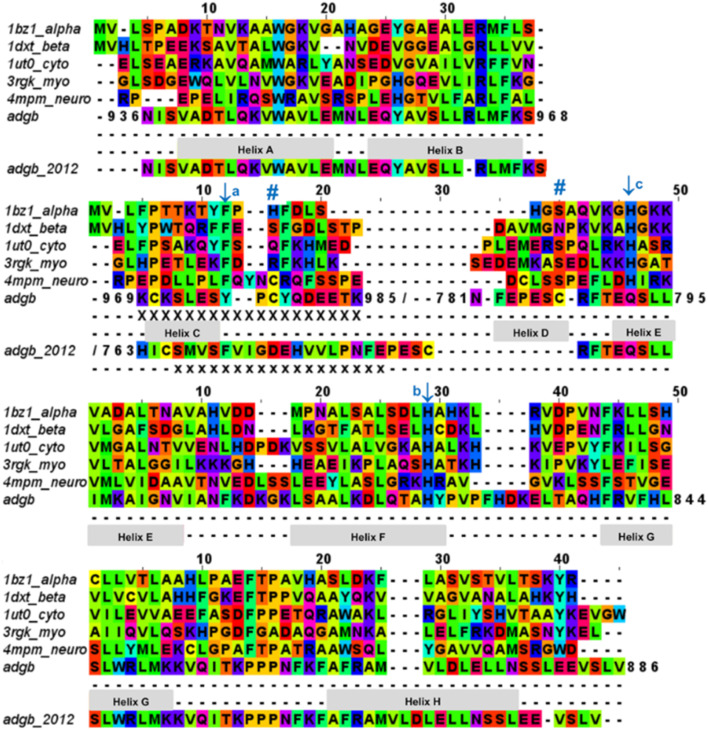
Androglobin heme-binding globin domain alignment. The alignment of the traditional globins was generated by structural alignment; the alignment of Adgb was determined using the in-house multi-template approach. The alignment denoted as Adgb_2012, previously reported by Hoogewijs *et al.*,^1^ is shown where it differs from our alternative proposed alignment. The six alignment zones coincide (bar helices C and D) with globin helices denoted as ‘Helix A’, ‘Helix B’ *etc.* as determined by inspection from the X-ray crystal structures. ‘/’ denotes the chain break in the discontiguous Adgb sequence, ‘XXX…’ denotes the extra residues used in the Adgb construct (this work) while ‘xxx…’ denotes the extra residues in the globin domain of Adgb reported by Hoogewijs *et al.*, 2012 (ref. 1), which here are identified by us as part of the preceding calpain C2-like domain (unpublished work). Key amino acids are denoted by labelled arrows, ‘a’ for the conserved CD1 loop aromatic residue, ‘b’ for the proximal histidine and ‘c’ for the distal histidine/glutamine; the disulfide bond is denoted by ‘#’.

Fig. 1 (left) shows the scores for various alternative alignments of the six key helices of Adgb-GD to their counterparts in alpha-Hb, beta-Hb, Cygb, Ngb, and Mb. Helix A (Adgb residues Val939-Glu951) is the first helix in alpha-Hb, beta-Hb, Cygb, Ngb and Mb, but in the naturally circular permuted Adgb it follows helix H (Adgb residues Phe866-Ser877) and the IQ domain (residues Val891-Thr933). The main peak lies at 0 for alpha-Hb, beta-Hb, Cygb, Ngb and Mb, which corresponds to the alignment in Fig. 2; a small alternative peak lies at +4 for each globin bar Mb, which corresponds to shifting the Adgb helix A four residues to the right. For the alpha-Hb–Adgb-GD alignment, there were 17 155 pairwise alignments (because there were 235 alpha-Hb sequences and 73 Adgb-GD sequences). When the Adgb sequences were moved between −25 and +25, the 0 alignment obtained 10 664 votes and the +4 alignment obtained 3664 votes; when the alpha-Hb sequences were moved between −25 and +25, the zero alignment obtained 10 001 votes and the +4 alignment obtained 774 votes. In this case, the mean of 10 332 for alignment 0 is considerably higher than the next nearest mean of 2219 (alignment +4). A similar predominant score was obtained for all HA alignments, and so when the scores for all five alignments were multiplied together, Fig. 1 (right), there is an overwhelming preference for alignment 0, despite the low percentage identity for HA of 15.3%, 15.2%, 16.6%, 21.1%, and 15.1% for the Adgb-GD alignments to alpha-Hb, beta-Hb, Cygb, Ngb, and Mb respectively. The results for the other five helices are similar, with percentage identities ranging between 7.8% (HH of the Adgb-GD–Ngb alignment) and 26% (HF of the Adgb–Mb alignment), Table S1.† Based on these percentages, Adgb-GD is marginally more similar to Mb (mean helical percentage identity 17.8%) and less similar to beta-Hb (mean helical percentage identity 14.0%). The full alignment is given in Fig. 2 and the mean percentage identity for the pairwise alignments for each helix–helix profile alignment between androglobin and the other hemoglobins is given in Table S1.†

The threefold Modeller structural alignment of both Adgb models (complex 1 and complex 2, see below) with the AlphaFold 2 model shows agreement in the sequence alignment except in two places, namely ^828^PVPFHDKEL^834^ (where helix F of the AlphaFold 2 model fills the space normally occupied by the heme) and the distal half of helix H, which terminates prematurely to accommodate the IQ domain (^872^DLWLLN^877^ is not helical).

The list of Adgb sequences, greatly expanded since the original sequence analysis in 2012, together with the helix alignment analysis above, permits a re-assessment of the overall sequence identification of the globin domain of Adgb. From the previous assignment of the globin domain structure,^1^ helices C to H are the first to be expressed on the N terminal side (His761 onwards). This is followed by C terminal helices A and B from Asp935 onwards. These sections are interconnected by a 33-amino acid section incorporating an IQ calmodulin binding domain (Fig. S1A†). This assignment took into account several key amino acids that are highly conserved throughout the hemoglobin superfamily. These comprised: (i) the proximal His connecting the heme iron to the protein, situated on the F helix (F8); (ii) the E7 distal heme iron ligand in Adgb, which is a Gln instead of the more common His residue;^1^ (iii) the CD1 Phe residue playing a role in anchorage and binding of the heme group within the heme pocket.^21,22^ While our sequence alignment agrees with most of the original alignment, the identities of the C helix region and the CD1 residue differ. With the Phe770 amino acid assignment as the CD1 residue in human Adgb, it becomes clear that in other species ∼22% of such alignments place a Cys in the CD1 position (Table S2†). Indeed, the previous alignment of Adgb globin domains shows three (9% of sequences) as Cys residues at the CD1 position,^1^ but this was interpreted as mismatched alignments. The adjusted sequence alignment, shown in Fig. S1B and C,† was used for the recombinant expression.

A Cys residue in the CD1 position is likely to have profound impact on the stabilization of heme binding as the substitution of the CD1 Phe by Cys removes an important contact with heme leaving a gap at the surface of the heme pocket which could result in instability. Mutations in human hemoglobin of CD1 Phe almost invariably result in instabilities in the protein, resulting in Heinz body formation, cyanosis and severe hemolytic anemia. A natural variation with a Cys residue in the CD1 position was found in the beta Hb chain of a Caucasian male infant (Hb Little Venice, β42[CD1] Phe → Cys).^23^ At 2 years of age, the infant showed severe chronic hemolytic anaemia, positive Heinz body formation, and haptoglobin depletion and required a monthly regular transfusion regime used more commonly for the cases of severe forms of thalassemia.^23^

We conducted a reassessment of the C to D helix sequence assignment, disregarding the prerequisite of a Phe as the CD1 component of the structure, based on the globin domain helix alignments (Fig. 1 and 2). Our assessment places a different sequence as the C to D helix section, thus placing a Tyr residue (Tyr976) in the CD1 position of the human Adgb globin domain (Fig. S1B and S2†). Although essentially unique in eukaryotes, the presence of a Tyr in the CD1 position of prokaryotic Hbs is common and does not significantly affect heme binding, but can affect oxygen binding affinity.^24^ Sequence alignment comparison shows that the frequency of Tyr residues is ∼82% with the majority of the remaining sequences being Phe residues (Table S2†). Therefore, we propose the assignment of CD1 to Tyr976 instead of Phe770, resulting in a circular permutation where helices D to H are expressed on the N-terminal side, followed by helices A to C following the IQ calmodulin binding domain sequence.

The previous alignment sequence did not allow expression of a stable form of the globin domain to be generated recombinantly,^10^ likely as a result of the expression of the incomplete globin domain. However, this new approach to sequence alignment resulted in expression of a stable globin domain (*vide infra*).

### The androglobin model structures

**Fig. 3:**
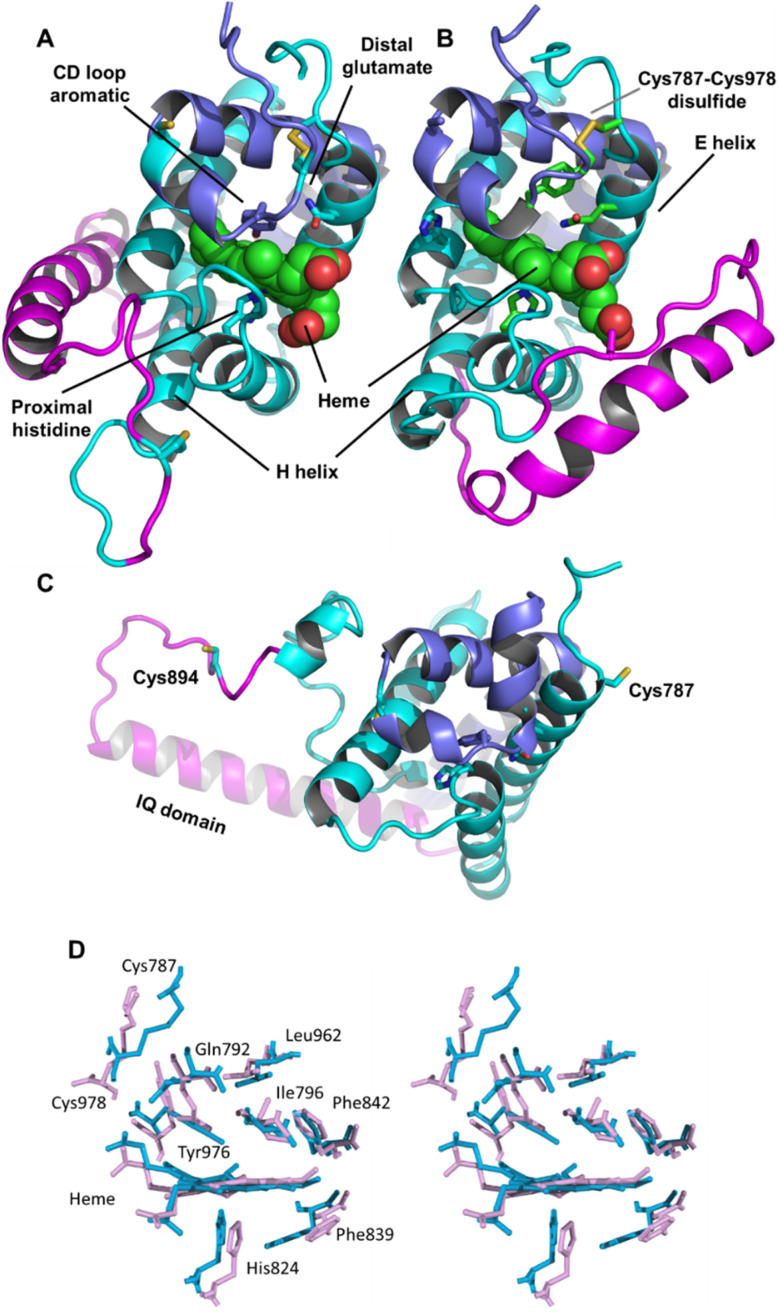
Structures of the heme-binding globin domain of androglobin. (A) The complex 1 structure determined using Modeller, (B) the complex 2 structure determined using Modeller and (C) the AlphaFold 2 structure. The structure is coloured blue for the N-terminus (helices D–H), magenta for the IQ domain insertion and cyan for the C terminus (helices A–C). (D) Stereo view of the predicted heme environment from complex 1 (blue) and complex 2 (purple).

Adgb-GD complexes 1 and 2, shown in Fig. 3, align well with the five Hb structural templates. The root mean square deviations (RMSDs) over the common globin domain to Mb are 1.3 Å for complex 1, 1.1 Å for complex 2 and 2.3 Å for the AlphaFold 2 model, over the alpha helices, as determined using SSM.^25^ The three models differ in the orientation of the IQ (calmodulin-binding) domain, which is not included in the RMSD calculations because it is absent in Mb and the other traditional Hbs. The orientation of the IQ domain differs in all three structures and is connected by a very flexible loop, and this flexibility is probably important for binding calmodulin. When superposed onto the full Adgb AlphaFold structure, all three structures present the IQ domain in an accessible orientation. The AlphaFold 2 and Modeller structures differ in two other aspects. Firstly, helix H is truncated in the AlphaFold 2 model. Secondly, helix E moves slightly into the space occupied by the heme; this structural topology may bear some resemblance to purified Adgb before the heme group is added back (see ESI†). The globin domain contains four cysteine residues, none of which form a disulfide bond in the AlphaFold 2 model.

**Fig. 4:**
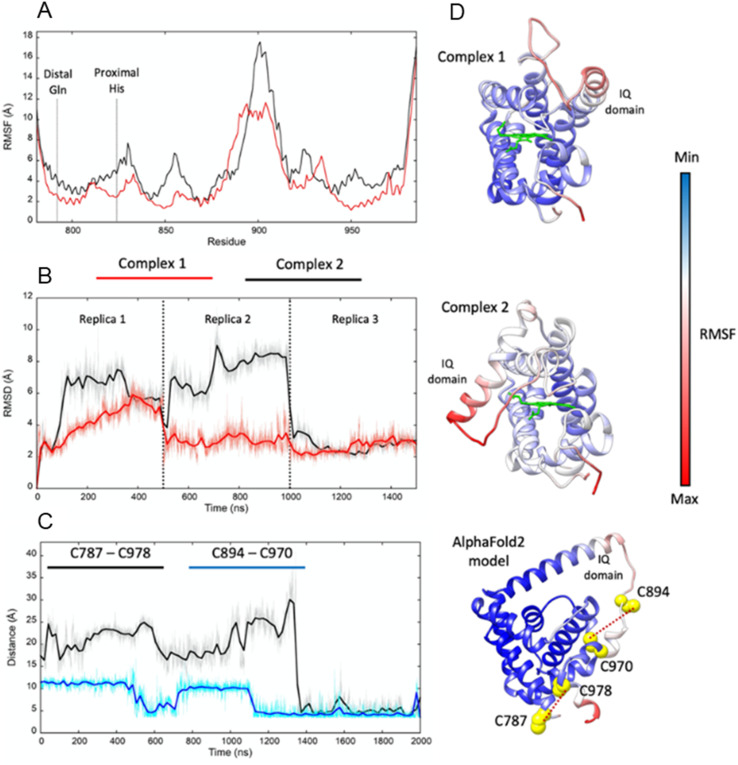
Molecular dynamics simulations of Adgb-GD complex 1, complex 2, and the AlphaFold 2 model. (A) RMSF comparison between complexes 1 (red) and 2 (black); (B) heme RMSD within complexes 1 and 2 during the MD replicas; (C) distances between pairs of cysteine residues during the MD simulation of the AlphaFold 2 model (C787–C978 in black and C894–C970 in blue); (D) RMSF plotted on complex 1, complex 2, and the AlphaFold 2 model (represented as a ribbon; heme is represented as a green stick); red ribbon colour indicates high structural flexibility; the four cysteine residues of the domain and their distance are shown on the AlphaFold 2 model.

Adgb-GD complexes 1 and 2 were investigated through molecular (MD) dynamics simulations to assess stability over the time course. Three 500 ns MD replicas were produced for each model in complex with the heme (Fig. 4). The emerging scenario suggests the overall higher stability of complex 1 as indicated by the root mean square fluctuation (RMSF) analysis (Fig. 4A and D) and the RMSD of the heme (Fig. 4B and D). In both structures, the IQ domain and the loop connecting it to the H helix were the most dynamic parts (ESI Videos 1 and 2†); however, in complex 1 the whole structure underwent high thermal fluctuations. This affected the stability of the heme: it was less steady in complex 1 (Fig. 4B). In both systems, the distal Gln792 remained in the proximity of the heme, while the CD Tyr976 was more flexible during the simulations, especially in complex 2. Models are accessible *via*https://zenodo.org/records/10509366.

### Optical properties, heme iron spin state and the intramolecular disulfide bond in the androglobin globin domain

SDS PAGE and western blot analysis show expression and purification of a soluble Adgb-GD protein at ∼26 kDa (Fig. S3†), close to the predicted 27 kDa weight based on the sequence as shown in Fig. S1C.† This includes the Histag and linker sequences and the alternative C-terminal C helix identified (*vide supra*). Reverse phase HPLC analysis of Adgb-GD shows the heme content used for extinction coefficient calculations as directly compared to a known concentration of Mb (Fig. S4†). Size exclusion analysis shows that in a phosphate buffer the protein is primarily monomeric with some dimer (Fig. S5†). In 150 mM NaCl buffer, this changed to mostly tetramer, likely as a result of the decrease of charge repulsion on the protein surface. All subsequent data were collected in the absence of salt unless otherwise stated. The predicted model of this domain (see ESI videos†) results in a large positive surface electrostatic potential for much of the protein. This may explain why SDS facilitates purification, *i.e.* prevents aggregation with endogenous *E. coli* proteins through blockage of charged sites.

**Fig. 5:**
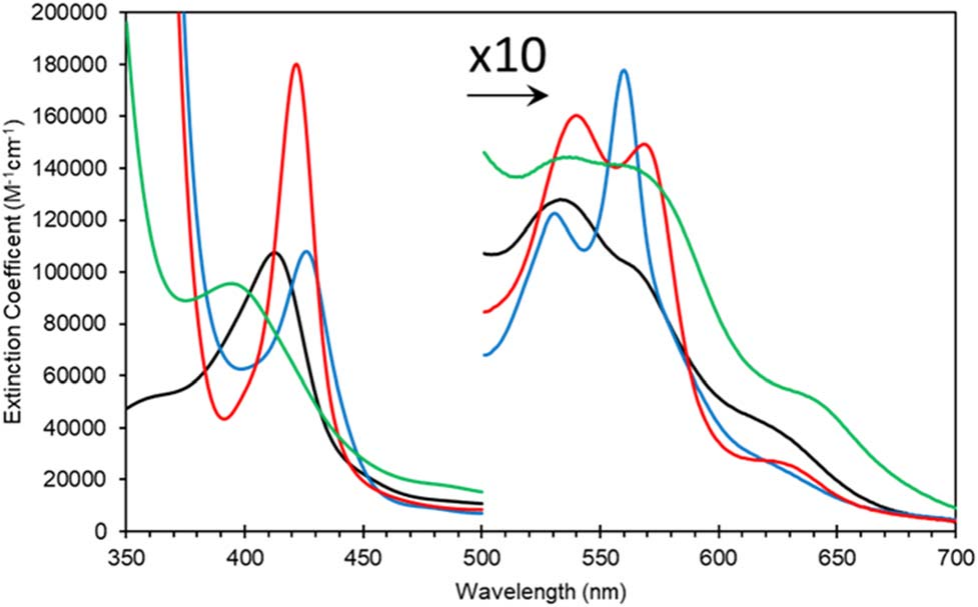
Optical characteristics of the androglobin globin domain. Protein was expressed based on revised amino acid assignment shown in Fig. 2 and S2.† Ferric (black line), dithionite reduced deoxy ferrous (blue line), ferrous–CO bound (red line) and ferrous–NO bound (green line).

As expressed, the optical properties of Adgb-GD are shown in Fig. 5 and the calculated extinction coefficient and peak wavelengths are in Table S3.† The ferric protein has bands in the visible region at 534 and 566 nm, suggesting a hexacoordinate state of the heme iron like that observed for Cygb and Ngb.^3,4^ However, a small peak at ∼630 nm suggests that the protein also has some pentacoordinate-like properties. The coordination is pH dependent as shown in Fig. S6.† There is a single transition with p*K*_a_ 8.6 that, from the optical spectra, is typical of a transition from penta- to hexa-coordination, with the loss of a 630 nm peak (typical of the water-bound pentacoordinate state) and the evolution of peaks at 566/534 nm.^26^ Mb and Hb have similar p*K*_a_ values (8.93 and 8.0 for Mb and Hb, respectively).^26^ However, the change in coordination is opposite to that of Adgb, with Adgb showing hexa-coordination at more acidic pH values and penta-coordination at alkaline pH. Thus the change in coordination cannot be due to the H_2_O/OH^−^ ligands observed in Mb and Hb and is more indicative of the coordination change observed with Cygb (pK 8.2) with His–Fe(iii)–X (X = His for Cygb and Gln for Adgb) at acidic pH and His–Fe(iii)–H_2_O at alkaline pH.^27^ Reduction to deoxy ferrous iron shows two prominent peaks at 531 and 560 nm indicative of hexacoordinate heme iron configuration. The CO-bound spectrum is typical for most globins, but the NO-bound spectrum exhibits a Soret peak at an unexpectedly hypsochromic (blue) shifted peak at 395 nm (Fig. 5). Typical wavelength maxima for hexacoordinate ferrous–NO bound protein are observed ∼420–430 nm in other NO-bound globins such as Mb and Cygb.^28,29^ This suggests that the NO is bound in an unusual form in Adgb-GD. Optical changes were not observed with addition of NO (20 mM) to ferric Adgb-GD, indicating that NO affinity for the ferric heme iron is low.

**Fig. 6:**
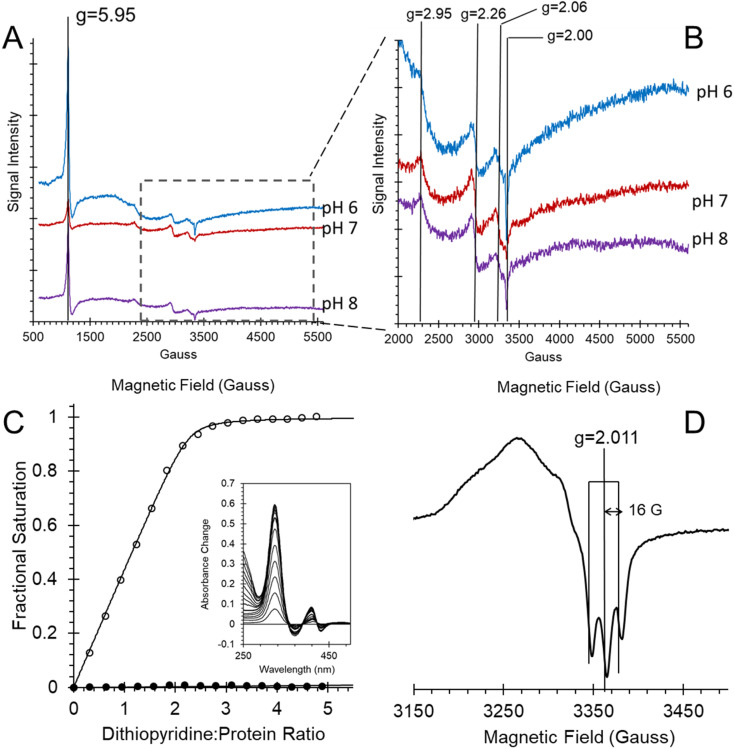
Androglobin heme iron ligation and cysteine oxidation states. (A) EPR spectra of the 80 μM ferric androglobin globin domain showing both high spin and low spin Fe^3+^ EPR signals at various pH values. The spectra were recorded at 10 K, 3.16 mW microwave power and 5 G modulation amplitude. (B) Expanded view of (A) highlighting the low spin signals. (C) Measurement of the surface exposed disulfide number and oxidation state. Protein (6.4 μM) was titrated with 4,4′-dithiodipyridine and the optical changes followed at 324 nm. The fractional saturation of dithiodipyridine binding to free sulfhydryl as a function of dithiodipyridine concentration for TCEP reduced (○) and purified forms (no disulfide reduction, ●) shows that one surface-exposed disulfide bond is present in the globin domain when expressed. Inset shows the optical changes of titration of TCEP reduced Adgb-GD with dithiodipyridine. (D) An EPR spectrum of ferrous NO bound Adgb-GD, exhibiting three lines, separated by 16 G, around *g* = 2.011, typical of other EPR spectra of five coordinate NO binding such as cyt c′. The spectrum was recorded at 10 K, 3.16 mW microwave power and 3 G modulation amplitude.

The EPR spectra of ferric heme Adgb-GD at a slightly acidic pH (Fig. 6A and B, pH 6) recorded at 10 K shows a mixture of high spin (*S* = 5/2, HS) and low spin (*S* = 1/2, LS) signals corresponding to penta- and hexa-coordinated heme iron, respectively. The HS signal with the perpendicular *g*_*x*_ = *g*_*y*_ = 5.95 and the parallel *g*_*z*_ = 2.00 components is typical of other globins in a pentacoordinate conformation. The *g* = 2.95 and *g* = 2.26 EPR signals are the *g*_*x*_ and *g*_*y*_ components of a LS signal, and the third *g*_*z*_ component was not observed as it was too broad, which is typical for many globins, particularly for the signal-to-noise level. The LS signal with these *g*-values has been classified as a low spin form in ferric Hb, with one of the axial ligands likely to be a histidine's nitrogen and the other not identified.^30^ At pH 7, the HS signal at *g* = 5.95 is smaller than at pH 6, which is concurrent with a noticeable increase of the LS form (as per both *g* = 2.95 and *g* = 2.26 signals). This is in agreement with the total ferric heme concentration being conserved in the pH 6–pH 7 range. The intensity of the HS peak partially returns at pH 8, with a small decrease of the LS form as compared to pH 7. However, this was likely not due to an acid–alkaline transition between the HS and LS ferric heme forms as the p*K*_a_ for such a transition is clearly reported in the liquid phase at 8.4 (Fig. S6†). The intensity changes are likely an artifact of freezing, similar to that observed with the EPR spectra of Cygb.^27^ This effect appears largely negated with the disulfide reduced protein where there was no effect of pH on both HS and LS signals (Fig. S7†). The reduced cysteine protein shows a change in the line shape of the perpendicular components area, at *g* ∼ 6. Not only does the *g* = 6 signal becomes much wider, the new effective *g*-values become apparent at *g* ∼ 6.5 (or maybe lower/higher). A small amount of ‘rhombic iron’ can be observed as a *g* = 4.3 signal in both oxidized and reduced proteins as a typical small component in native and recombinant proteins.^31^

In Ngb, the CD loop contains a disulfide bond between Cys46 (helix C7) and Cys55 (helix D5), known to stabilize the distal histidine ligation and the redox thermodynamics of ferric Ngb.^32–34^ With our reassignment of the C helix sequence, the CD section of Adgb-GD possesses a predicted disulfide bond from residues Cys787 and Cys978 (Fig. S1†). Absent in the original sequence alignment, this disulfide bond could stabilize this crucial juncture of the heme pocket, now predicted as the N and C terminal sections of the domain. With four cysteine residues in the globin domain sequence, we determined how many of those are surface exposed and free to bind dithiodipyridine. As shown in Fig. 6C, there were two (1.9 ± 0.3) free sulfhydryls per heme detected with tris(2-carboxyethyl)phosphine (TCEP) reduced Adgb-GD, meaning that two of the four cysteines are surface exposed. In the protein as expressed in *E. coli* (without reduction by TCEP), no free cysteines were observed. Thus, as expressed, the globin domain possesses a single disulfide bond. From the predicted positions of the cysteines in complexes 1 and 2, only two are both surface exposed and close enough to form an intramolecular disulfide, which are Cys787 and 978 in the “CD loop” region of the protein; however, both these two (reduced) cysteine residues Cys894 and Cys970 moved sufficiently close to form a disulfide bond in the MD simulation of the AlphaFold 2 structure after 1.4 μs (Fig. 4C), even though the sulfur atoms were originally 11.6 Å and 17.4 Å apart respectively (Fig. 4D). Further studies will be required to confirm the position and the micro-environmental conditions favoring the formation of the disulfide bond within Adgb-GD.

### Ferrous androglobin globin domain binds nitric oxide in a primarily pentacoordinate form

In order to further establish that the heme group is fully incorporated into a heme pocket, as in other heme proteins and not merely adventitiously bound to the surface, we have studied the ligand binding properties of Adgb-GD. By using stopped-flow and femto-second laser spectroscopy, we have shown that the heme behaves as expected for a fully incorporated heme.

Adgb-GD binds NO in the ferrous form to generate an optical spectrum as observed in Fig. 5, suggesting a five-coordinate NO heme iron. This is supported by EPR at 10 K (Fig. 6D). This spectrum showed a characteristic three-line hyperfine signal, essentially identical to that reported for cyt c′ from *Shewanella frigidimarina* and *Alcaligenes xylosoxidans*.^16^ This is again consistent with five-coordinate NO binding to the ferrous heme.^16,35^

The optical changes and kinetics of NO binding to deoxyferrous Adgb-GD, followed using stopped-flow spectroscopy, are shown in Fig. S8.† Increases at 390 nm are concurrent with decreases at 426 nm with a number of isosbestic points (*e.g.* 407 nm) consistent with a simple Fe^2+^ + NO → Fe^2+^–NO binding mechanism. However, the time course follows a double exponential function (Fig. S8C, E and F†), suggesting either a heterogeneous population or an intermediate that is distinct from the NO-bound or deoxyferrous species. A heterogenous population is supported by a global fit to a 3-component parallel or serial mechanism showing essentially identical calculated component spectra; the starting deoxyferrous, the end NO-bound and a spectrum that is intermediate between the deoxyferrous species and ferrous–NO species with clear isosbestic points (Fig. S8D†). This eliminates the possibility that the slow phase results from a displacement of a bound ligand by NO as optically distinct intermediates would be expected. This suggests that a conformational change from a closed to an open form of the protein may be required for NO binding.

The rate constant for NO binding is NO concentration independent for both phases (Fig. S8E and F†), suggesting that there two populations of the heme pocket, both having an occluded (closed) heme where a non-binding ligand sterically hindered the approach of NO. The rate limits observed are thus the rate constants of the conformational change in each population for the closed to open transition. The oxidation state of the cysteines makes no difference to the rate of NO binding, either the fast or the slow phase. Therefore, any effect of the cysteine oxidation state on heme pocket dynamics does not hinder the entrance, binding and dissociation of NO to the heme iron. An estimate of the maximum rate of binding may be made from Fig. S8E and F,† at concentrations of NO less than 12 μM. The affinity of NO binding is measured to be ∼1 × 10^−10^ to 1 × 10^−11^ M (Table S4†) with *k*_on_ values ≥ 10^7^ and 10^6^ M^−1^ s^−1^ based on the lowest concentration of NO used and *k*_off_ calculated from ligand exchange with oxygen (1.6 × 10^−4^ s^−1^, Fig. S9†). This affinity is consistent with other globins such as Mb, Hb and Ngb where *k*_on_ is ∼10^7^ to 10^8^ M^−1^ s^−1^ and *k*_off_ 10^−3^ to 10^−5^ s^−1^.^36,37^

CO binding exhibits a similar kinetic pattern to NO binding, albeit at slower rates (Fig. S10†). At the concentrations used the CO binding was CO concentration independent, like that of NO. However, unlike NO binding the reduction of the cysteines did show an effect on CO binding, with a decrease in the kinetics of CO binding of ∼3.5 fold (Fig. S10E and F†). The affinity of the CO binding is ∼10^−7^ to 10^−8^ M (Table S4†) with fast and slow *k*_on_ values ≥ 3 × 10^6^ and 3 × 10^5^ M^−1^ s^−1^ respectively based on the lowest concentration of CO used and *k*_off_ on ligand exchange with NO (0.31 s^−1^, Fig. S11†). This affinity is ∼6000 fold lower than NO binding, consistent with the ‘sliding scale rule’ developed by Olson and coworkers^38^ and is consistent with other globins such as Mb and Ngb where *k*_on_ is ∼10^7^ to 10^8^ M^−1^ s^−1^ and *k*_off_ 10^−3^ to 10^−5^ s^−1^.^38,39^

The five-coordinate NO-bound species found in this work is similar to those reported for cyt c′, with the NO on the proximal side of the heme. The kinetics of NO binding to deoxyferrous Adgb-GD (Fig. S8†) is consistent with the NO binding mechanism proposed for cyt c′, that is a single optical transition comprising two kinetic phases.^16^ NO can only bind to the distal iron location of cyt c′ following a conformational change involving an occlusion in the heme pocket (by a Phe residue in the case of cyt c′). These two conformations of cyt c′ are optically indistinguishable. Subsequent to proximal histidine displacement, NO binding to the proximal side of the heme and NO dissociation from the distal side are rapid and not observed as separate events, but only as a single observed spectrokinetic event. This model may also be applied to the observations of NO binding to Adgb-GD. However, there is insufficient evidence to assign whether NO binds to the proximal or distal side, although a simple hypothesis is that it binds to the distal side. Furthermore, cyt c′ shows a mixture of penta- and hexa-coordinate NO bound species as observed by a split Soret peak 395/415 nm.^40^ As a weak band is consistently present in NO binding and NiR experiments at ∼420–430 nm, it is likely that a small fraction of NO-bound Adgb-GD remains as a hexa-coordinated species.

In addition to cyt c′, Hb has also been reported to bind NO in a pentacoordinate form, but only in a T state at low NO to Hb ratios and only with the alpha chain.^33,41,42^

**Fig. 7:**
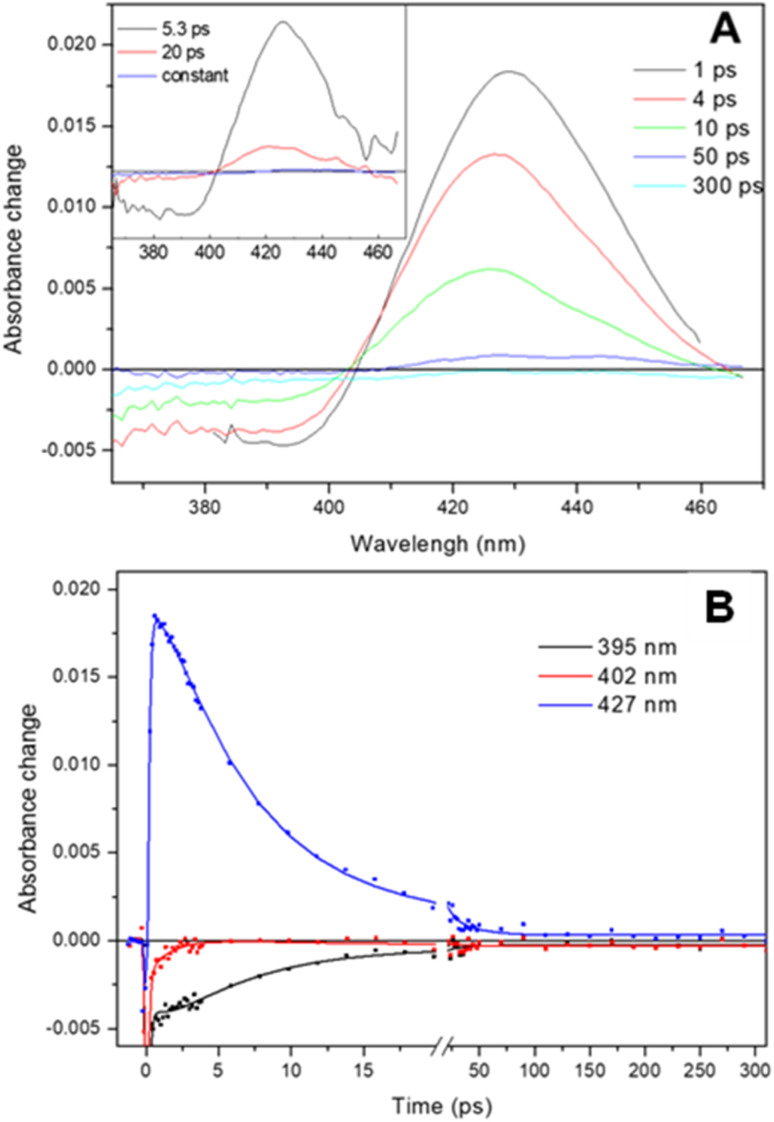
Ultrafast photo-dissociation and rebinding of NO from the ferrous Adgb-GD five coordinate NO complex. (A) Transient absorption spectra after different delay times upon excitation at 570 nm. (Inset) Decay associated spectra corresponding to the NO geminate rebinding phases obtained from a global analysis and (B) dual-timescale kinetics and fits at selected wavelengths.

The transient absorption spectra observed following dissociation of the Adgb-GD : NO complex resulting from a short (femtosecond) light pulse are shown in Fig. 7A. The spectra are characterized by a broad bleaching around 390 nm due to the disappearance of the 5-coordinate NO-bound state and a relatively strong induced absorption centered at 427 nm assigned to the 4-coordinate NO-dissociated state.^43,44^ This supports the EPR data (Fig. 6D) showing that the NO bound state of the ferrous Adgb is pentacoordinate. Apart from small relaxation signals with a time constant of ∼1.5 ps, corresponding to a blue shift of the induced absorption band (Fig. 7A inset) and assigned to vibrational cooling, the spectral evolution is characterized by a decay (associated spectra in the inset of Fig. 7A) dominated by a 5.3 ps phase (1.9 × 10^11^ s^−1^) and a minor (∼14%) 20 ps phase (5.0 × 10^10^ s^−1^) (Fig. 7B). The remaining spectrum after these phases corresponds to only ∼1% of the photo-dissociated NO, meaning that NO rebinding is almost completely geminate. This implies that dissociated NO stays within the confines of the heme pocket and only minor quantities of NO escape the heme pocket. Rebinding of NO to the heme iron from bulk solution outside the heme pocket would be expected to occur on the μs to ms timescale, as observed with Mb and Cygb for NO or other gases such as CO.^29,45,46^ High-yield rebinding of NO to heme in a single 5–8 ps phase has been observed upon dissociation in all studied 5-coordinate heme-NO complexes in proteins thus far (Table S5†).^47–49^ In Adgb, however, a slower, 20 ps phase of NO binding is also present. This finding suggests a relaxation process competing with initial NO rebinding, allowing NO to explore a larger conformational space (rototranslational freedom) and indicating a less constrained heme pocket. Such multiphasic NO rebinding dynamics reflecting a more accessible heme pocket is also observed in other globins, which all form 6-coordinate NO complexes (Table S5†).

### Androglobin nitrite reductase activity and effect of the cysteine redox state on behaviour

**Fig. 8:**
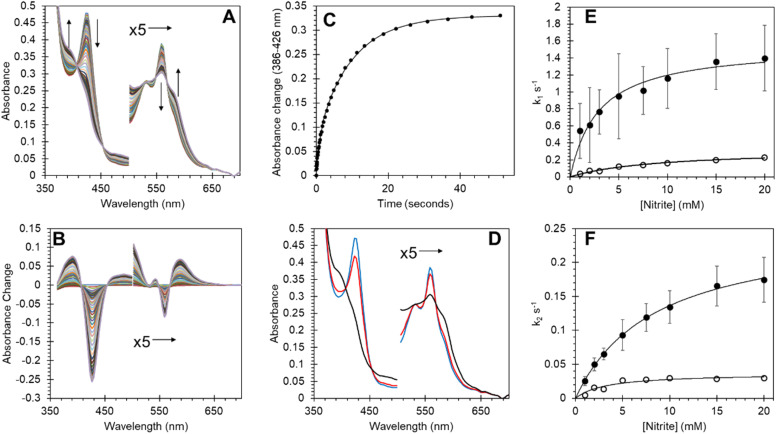
Nitrite reductase activity of Adgb-GD and effect of the disulfide redox state on reductase activity. (A) Optical spectra of deoxyferrous Adgb-GD (5 μM) with sodium nitrite (5 mM). (B) Difference spectra with initial ferrous protein set to zero. (C) Time course of optical changes fitted to a double exponential function (*k*_1_ = 9.25 × 10^−1^ s^−1^ and *k*_2_ = 1.02 × 10^−1^ s^−1^). (D) Global fit of initial deoxyferrous protein (blue), intermediate (red) and final ferrous–NO bound spectra (black). (E and F) The dependence of the rate constants for Adgb nitrite reductase activity. The observed rate constants of the fast (E) and slow (F) phases on the reaction in the presence (●) or absence (by reduction using TCEP, ○) of the CD disulfide bond.

Many heme proteins, including Hbs, are noted for their nitrite reductase (NiR) activity, giving potential functions in NO homeostasis depending on the oxygen concentration of their microenvironment.^29,50–52^ This reaction was assessed for Adgb-GD under anaerobic conditions (Fig. 8). The reaction follows a mechanism in which NO_2_^−^ slowly reacts with ferrous heme to generate NO and ferric heme.^53,54^ The ferric heme is rapidly reduced by excess dithionite in solution resulting in the formation of a ferrous–NO complex. The optical changes following this reaction are essentially identical to those of NO binding (Fig. 8A and B; compare with Fig. S8A and B†) with two-phase kinetics (Fig. 8C). The concentration dependence of the fast kinetics of the nitrite reaction (Fig. 8E) exhibits a high error due to the small amplitude of the optical changes observed in the global fit (Fig. 8D) but appears to follow a hyperbolic concentration dependence with an apparent *K*_D_ of 2.91 ± 0.57 mM NO_2_^−^ and a maximum rate of 1.54 s^−1^ ± 0.01 s^−1^ (Fig. 8E, ●). The slower rate representing the formation of the deoxyferrous–NO bound species also follows a hyperbolic curve as a function of nitrite concentration with an apparent *K*_D_ of 8.42 ± 0.46 mM NO_2_^−^ and a maximum rate of 2.54 × 10^−1^ s^−1^ ± 6.1 × 10^−4^ s^−1^ (Fig. 8F, ●). Unlike NO binding, the reduction of the disulfide bond has a significant effect on the rate of NiR activity (Fig. 8E and F) with TCEP reduced free sulfhydryl (○) exhibiting significantly decreased kinetics, both for the fast kinetics with an apparent *K*_D_ of 9.13 ± 1.38 mM NO_2_^−^ and a maximum rate of 3.23 × 10^−1^ s^−1^ ± 0.02 s^−1^ and slower NO binding kinetics with an apparent *K*_D_ of 3.26 ± 1.12 mM NO_2_^−^ and a maximum rate of 3.67 ± 0.40 × 10^−2^ s^−1^. This effect of sulfhydryl reduction on the NiR activity may arise from structural changes in the heme pocket, affecting exogenous ligand affinity, as observed for Ngb,^34^ or affecting the endogenous distal ligand off-rate, as observed for Ngb^54^ and Cygb.^45^

The oxyform which can only be observed transiently by stopped-flow spectroscopy binds with an association rate constant of ∼0.8 × 10^6^ M^−1^ s^−1^. This compares to 14, 50, 60 and 250 × 10^6^ M^−1^ s^−1^ for Mb, Hb α, Hb β and Ngb, respectively.^55,56^ Autoxidation rates for globins vary greatly, with Mb and Ngb reported at 0.055 and 5.4 h^−1^.^55^ The measured autoxidation rate for Adgb-GD is ≥2.6 s^−1^ (9360 h^−1^) being limited by the oxidation of dithionite. This value is three orders of magnitude higher than the corresponding value for Ngb (Fig. S12†). This remarkable autoxidation rate strongly suggests that O_2_ binding is not a physiological function of the protein except for the possibility of oxygen sensing. Similarly, NO dioxygenase activity is unlikely, as this requires a semi-stable ferrous–O_2_ complex for the NO to react with. The oxidation state of the iron *in vivo* remains unknown, like that of Ngb and Cygb. However, the oxygen tension in the testes is typically low (∼10–15 mmHg), lower even than venal O_2_ levels.^57–59^ Consequently the existence of the ferrous form of the heme iron *in vivo*, at least in the testes, cannot be completely discounted.

Recent studies have shown that many globins appear to have properties relating to NO homeostasis, although the exact nature of this biochemistry *in vivo* is still under debate. Our results in Fig. 8 confirm that Adgb-GD reacts with nitrite to generate NO under hypoxic/anoxic conditions and subsequently binds NO as a pentacoordinate species. The NiR rate constants of other globins are typically linear as a function of nitrite concentration.^29^ Assuming that the rate of NO formation relates to the rate of ferrous–NO generation (Fig. 8F), the initial slope of ∼25 M^−1^ s^−1^ at low nitrite concentrations is higher than that for Ngb, Mb or Hb, and similar to that reported for globin X^50^ and Cygb (Table S6†).^29,60^

NO has important roles in the testes and hence NO homeostasis is also important. Four Nitric Oxide Synthases (NOS) are present in the testes, endothelial NOS (eNOS), inducible NOS (iNOS), and neuronal NOS (nNOS) and testis-specific NOS (TnNOS).^17^ NO has been proposed to play a unique role in modulating germ cell viability and development,^20^ with high NO concentrations exhibiting a deleterious role in the mobility of spermatozoa^61^ and thus acting on some aspects of male infertility.^17^ NO also has vital roles in steroidogenesis, gametogenesis and the regulation of germ-cell apoptosis with excess NO linked to induction of germ cell apoptosis and oxidative damage.^62^ Many globins have proven or proposed functions in NO regulation, either in primary or secondary roles. Thus, there is a distinct possibility that Adgb could also play a role in NO homeostasis and hence germ cell viability. Our data showing an unusual NO binding and high NiR activity provide a proof-of-principle that Adgb could be involved in this process, but it requires further studies to verify.

The presence of a calpain-like motif on the N terminal side of the globin domain and a calmodulin binding domain directly dissecting the globin domain strongly suggests a role for calcium in the functional mechanism of this protein. The links between NO and calcium are well established.^63^ Calcium channel blockers decrease intracellular calcium levels and increase the vasodilator efficacy of NO in smooth muscle.^64^ Conversely, an increase in NO by vascular endothelial cells in the liver enhances calcium signaling in surrounding hepatocytes.^65^ In the testes, calcium channel blockers to relieve hypertension causes reversible male infertility in mice.^66^ Hence it is not implausible that calcium and NO binding are linked to Adgb function.

Recently, the establishment of a role for Adgb in ciliogenesis has heightened the importance of Adgb. Overexpression studies show an Adgb-dependent increase in ciliated cells.^8^ The expression of Adgb is in turn linked to Forkhead Box J1 (FOXJ1), a transcription factor involved in ciliogenesis, and as overexpression of FOXJ1 directly led to increased Adgb mRNA levels through binding to the ADGB promotor.^8^ Furthermore, Adgb has been shown to directly interact with the cytoskeletal protein Sept10.^9^ Adgb knockout in mice leads to mislocalisation of Sept10 in sperm, resulting in malformations of the flagella and head. The mechanism of Adgb action appears to be *via* proteolytic cleavage of Sept10, with the proteolytic activity controlled *via* calmodulin-dependent binding to the IQ motif of the circularly permuted globin domain of Adgb.^9^

## Conclusions

In summary, the helical alignment for Adgb-GD from our method, designed to work in the twilight zone, yielded an alternate helix alignment around the C and D helical regions. This alignment is consistent with that obtained from the AlphaFold-2 model. The identification of a Tyr in the CD1 position (Tyr977 in the human Adgb) is to our knowledge unique for eukaryotic globins, but is common in prokaryotes.

The validation of our proposed helix alignment lies, at least in part, in the agreement with the AlphaFold-2 model and the generation of a stable recombinant form of the protein using the alternative globin domain sequence. This is supported by heme insertion and tight heme binding to generate optical and EPR spectra, typical of pure authentic heme proteins. The femtosecond laser flash data indicate a true heme pocket from which ligands (NO) cannot escape and kinetics measured by stopped-flow spectroscopy that conform in general to known heme proteins. The presence of an intramolecular disulfide bond goes some way to explain the stability of the recombinant protein, given that the N and C terminal regions of this circularly permuted globin domain are in the critical area of the heme-binding pocket.

The biochemical evaluation presented here illustrates the characteristics of the globin domain of Adgb, showing that it can potentially participate in NO sensing or regulation. Our results, together with the known calcium-linked structural aspects of the calpain and calmodulin binding properties of Adgb, lay the foundation for further investigation into the functional role of Adgb, given its potential medical significance.

## Data availability

Molecular simulation models have been deposited as videos and are accessible *via*https://zenodo.org/records/10509366. Additional experimental details and data are provided in the ESI,† including pairwise alignments for each helix–helix profile alignment, globin domain sequence alignments, frequency analysis of amino acid CD1 assignment, a comparison of globin domain optical wavelength maxima, extinction coefficients, ligand binding constants, geminate recombination binding constant and nitrite reductase activity with other globins, SDS-PAGE and immunoblot analysis of globin domain purity, heme content analysis, oligomer analysis, ferric heme acid–alkaline transition, cysteine reduced EP data, NO and CO binding spectra and kinetic data, O_2_–NO and NO–CO ligand exchange data and autoxidation data.

## Author contributions

BJR and CAR conceived the study, and BJR, JU and MTW performed the biochemical analysis of the protein and wrote the paper. CAR, CR, JCM, SA, and GD conducted the sequence, helix alignment and molecular dynamics studies, analysed the data and wrote the paper. CAR and CR conduced the helix alignments. EW and JA performed supplementary biochemical data collection and analysis for size exclusion (EW) and SDS PAGE/western blot (JA). DAS, BJR and JA performed and analysed the EPR study. MHV performed and analysed the ultra-fast laser flash study.

## Conflicts of interest

There are no conflicts to declare.

## Supplementary Material

SC-015-D4SC00953C-s001

SC-015-D4SC00953C-s002

SC-015-D4SC00953C-s003

## References

[cit1] Hoogewijs D., Ebner B., Germani F., Hoffmann F. G., Fabrizius A., Moens L., Burmester T., Dewilde S., Storz J. F., Vinogradov S. N., Hankeln T. (2012). Androglobin: a chimeric globin in metazoans that is preferentially expressed in Mammalian testes. Mol. Biol. Evol..

[cit2] Perutz M. F. (1960). Structure of hemoglobin. Brookhaven Symp. Biol..

[cit3] Trent III J. T., Watts R. A., Hargrove M. S. (2001). Human neuroglobin, a hexacoordinate hemoglobin that reversibly binds oxygen. J. Biol. Chem..

[cit4] Burmester T., Ebner B., Weich B., Hankeln T. (2002). Cytoglobin: a novel globin type ubiquitously expressed in vertebrate tissues. Mol. Biol. Evol..

[cit5] Zhu H., Riggs A. F. (1992). Yeast flavohemoglobin is an ancient protein related to globins and a reductase family. Proc. Natl. Acad. Sci. U. S. A..

[cit6] Rawlings N. D. (2015). Bacterial calpains and the evolution of the calpain (C2) family of peptidases. Biol. Direct.

[cit7] Huang B., Lu Y. S., Li X., Zhu Z. C., Li K., Liu J. W., Zheng J., Hu Z. L. (2014). Androglobin knockdown inhibits growth of glioma cell lines. Int. J. Clin. Exp. Pathol..

[cit8] Koay T. W., Osterhof C., Orlando I. M. C., Keppner A., Andre D., Yousefian S., Suarez Alonso M., Correia M., Markworth R., Schodel J., Hankeln T., Hoogewijs D. (2021). Androglobin gene expression patterns and FOXJ1-dependent regulation indicate its functional association with ciliogenesis. J. Biol. Chem..

[cit9] Keppner A., Correia M., Santambrogio S., Koay T. W., Maric D., Osterhof C., Winter D. V., Clerc A., Stumpe M., Chalmel F., Dewilde S., Odermatt A., Kressler D., Hankeln T., Wenger R. H., Hoogewijs D. (2022). Androglobin, a chimeric mammalian globin, is required for male fertility. Elife.

[cit10] Bracke A., Hoogewijs D., Dewilde S. (2018). Exploring three different expression systems for recombinant expression of globins: Escherichia coli, Pichia pastoris and Spodoptera frugiperda. Anal. Biochem..

[cit11] Lock A., Forfar R., Weston C., Bowsher L., Upton G. J., Reynolds C. A., Ladds G., Dixon A. M. (2014). One motif to bind them: A small-XXX-small motif affects transmembrane domain 1 oligomerization, function, localization, and cross-talk between two yeast GPCRs. Biochim. Biophys. Acta.

[cit12] Taddese B., Upton G. J., Bailey G. R., Jordan S. R., Abdulla N. Y., Reeves P. J., Reynolds C. A. (2014). Do plants contain g protein-coupled receptors?. Plant Physiol..

[cit13] Watkins H. A., Chakravarthy M., Abhayawardana R. S., Gingell J. J., Garelja M., Pardamwar M., McElhinney J. M., Lathbridge A., Constantine A., Harris P. W., Yuen T. Y., Brimble M. A., Barwell J., Poyner D. R., Woolley M. J., Conner A. C., Pioszak A. A., Reynolds C. A., Hay D. L. (2016). Receptor Activity-modifying Proteins 2 and 3 Generate Adrenomedullin Receptor Subtypes with Distinct Molecular Properties. J. Biol. Chem..

[cit14] Cutruzzola F., Arcovito A., Giardina G., della Longa S., D'Angelo P., Rinaldo S. (2014). Distal-proximal crosstalk in the heme binding pocket of the NO sensor DNR. BioMetals.

[cit15] Lawson D. M., Stevenson C. E., Andrew C. R., Eady R. R. (2000). Unprecedented proximal binding of nitric oxide to heme: implications for guanylate cyclase. EMBO J..

[cit16] Manole A., Kekilli D., Svistunenko D. A., Wilson M. T., Dobbin P. S., Hough M. A. (2015). Conformational control of the binding of diatomic gases to cytochrome c'. J. Biol. Inorg. Chem..

[cit17] Lee N. P., Cheng C. Y. (2008). Nitric oxide and cyclic nucleotides: their roles in junction dynamics and spermatogenesis. Adv. Exp. Med. Biol..

[cit18] Sataric M. V., Zdravkovic S., Nemes T., Sataric B. M. (2020). Calcium signaling modulates the dynamics of cilia and flagella. Eur. Biophys. J..

[cit19] Saternos H. C., AbouAlaiwi W. A. (2018). Signaling interplay between primary cilia and nitric oxide: A mini review. Nitric Oxide.

[cit20] Zini A., O'Bryan M. K., Magid M. S., Schlegel P. N. (1996). Immunohistochemical localization of endothelial nitric oxide synthase in human testis, epididymis, and vas deferens suggests a possible role for nitric oxide in spermatogenesis, sperm maturation, and programmed cell death. Biol. Reprod..

[cit21] Hargrove M. S., Krzywda S., Wilkinson A. J., Dou Y., Ikeda-Saito M., Olson J. S. (1994). Stability of myoglobin: a model for the folding of heme proteins. Biochemistry.

[cit22] Roesner A., Fuchs C., Hankeln T., Burmester T. (2005). A globin gene of ancient evolutionary origin in lower vertebrates: evidence for two distinct globin families in animals. Mol. Biol. Evol..

[cit23] Henderson S. J., Timbs A. T., McCarthy J., Gallienne A. E., Proven M., Rugless M. J., Lopez H., Eglinton J., Dziedzic D., Beardsall M., Khalil M. S., Old J. M. (2016). Ten Years of Routine alpha- and beta-Globin Gene Sequencing in UK Hemoglobinopathy Referrals Reveals 60 Novel Mutations. Hemoglobin.

[cit24] Ouellet H., Milani M., LaBarre M., Bolognesi M., Couture M., Guertin M. (2007). The roles of Tyr(CD1) and Trp(G8) in Mycobacterium tuberculosis truncated hemoglobin O in ligand binding and on the heme distal site architecture. Biochemistry.

[cit25] Mitchell E. M., Artymiuk P. J., Rice D. W., Willett P. (1990). Use of Techniques Derived from Graph-Theory to Compare Secondary Structure Motifs in Proteins. J. Mol. Biol..

[cit26] Antonini E. and Brunori M., in Frontiers in Biology, ed. A. Neuberger and E. L. Tatum, North-Holland Publishing Company, Amsterdam-London, 1971, vol. 21, pp. 13–52

[cit27] Reeder B. J., Svistunenko D. A., Wilson M. T. (2011). Lipid binding to cytoglobin leads to a change in haem co-ordination: a role for cytoglobin in lipid signalling of oxidative stress. Biochem. J..

[cit28] Li H., Hemann C., Abdelghany T. M., El-Mahdy M. A., Zweier J. L. (2012). Characterization of the mechanism and magnitude of cytoglobin-mediated nitrite reduction and nitric oxide generation under anaerobic conditions. J. Biol. Chem..

[cit29] Reeder B. J., Ukeri J. (2018). Strong modulation of nitrite reductase activity of cytoglobin by disulfide bond oxidation: Implications for nitric oxide homeostasis. Nitric Oxide.

[cit30] Blumberg W. E. and Peisach J., in Probes of structure and function of macromolecules and membranes, ed. B. Chance, T. Yonetani and A. S. Mildvan, Acedemic Press, New York, 1971, vol. 2, pp. 215–229

[cit31] Svistunenko D. A., Reeder B. J., Wankasi M. M., Silaghi-Dumitrescu R. L., Cooper C. E., Rinaldo S., Cutruzzola F., Wilson M. T. (2007). Reaction of Aplysia limacina metmyoglobin with hydrogen peroxide. Dalton Trans..

[cit32] Bellei M., Bortolotti C. A., Di Rocco G., Borsari M., Lancellotti L., Ranieri A., Sola M., Battistuzzi G. (2018). The influence of the Cys46/Cys55 disulfide bond on the redox and spectroscopic properties of human neuroglobin. J. Inorg. Biochem..

[cit33] Hamdane D., Kiger L., Dewilde S., Green B. N., Pesce A., Uzan J., Burmester T., Hankeln T., Bolognesi M., Moens L., Marden M. C. (2004). Coupling of the heme and an internal disulfide bond in human neuroglobin. Micron.

[cit34] Vinck E., Van Doorslaer S., Dewilde S., Moens L. (2004). Structural change of the heme pocket due to disulfide bridge formation is significantly larger for neuroglobin than for cytoglobin. J. Am. Chem. Soc..

[cit35] Zhao Y., Hoganson C., Babcock G. T., Marletta M. A. (1998). Structural changes in the heme proximal pocket induced by nitric oxide binding to soluble guanylate cyclase. Biochemistry.

[cit36] Brunori M., Giuffre A., Nienhaus K., Nienhaus G. U., Scandurra F. M., Vallone B. (2005). Neuroglobin, nitric oxide, and oxygen: functional pathways and conformational changes. Proc. Natl. Acad. Sci. U. S. A..

[cit37] Cooper C. E. (1999). Nitric oxide and iron proteins. Biochim. Biophys. Acta.

[cit38] Tsai A. L., Berka V., Martin E., Olson J. S. (2012). A “sliding scale rule” for selectivity among NO, CO, and O(2) by heme protein sensors. Biochemistry.

[cit39] Uzan J., Dewilde S., Burmester T., Hankeln T., Moens L., Hamdane D., Marden M. C., Kiger L. (2004). Neuroglobin and other hexacoordinated hemoglobins show a weak temperature dependence of oxygen binding. Biophys. J..

[cit40] Adams H. R., Svistunenko D. A., Wilson M. T., Fujii S., Strange R. W., Hardy Z. A., Vazquez P. A., Dabritz T., Streblow G. J., Andrew C. R., Hough M. A. (2023). A heme pocket aromatic quadrupole modulates gas binding to cytochrome c'-beta: Implications for NO sensors. J. Biol. Chem..

[cit41] Fago A., Crumbliss A. L., Peterson J., Pearce L. L., Bonaventura C. (2003). The case of the missing NO-hemoglobin: spectral changes suggestive of heme redox reactions reflect changes in NO-heme geometry. Proc. Natl. Acad. Sci. U. S. A..

[cit42] Yonetani T., Tsuneshige A., Zhou Y., Chen X. (1998). Electron paramagnetic resonance and oxygen binding studies of alpha-Nitrosyl hemoglobin. A novel oxygen carrier having no-assisted allosteric functions. J. Biol. Chem..

[cit43] Negrerie M., Bouzhir L., Martin J. L., Liebl U. (2001). Control of nitric oxide dynamics by guanylate cyclase in its activated state. J. Biol. Chem..

[cit44] Silkstone G., Kapetanaki S. M., Husu I., Vos M. H., Wilson M. T. (2010). Nitric oxide binds to the proximal heme coordination site of the ferrocytochrome c/cardiolipin complex: formation mechanism and dynamics. J. Biol. Chem..

[cit45] Beckerson P., Reeder B. J., Wilson M. T. (2015). Coupling of disulfide bond and distal histidine dissociation in human ferrous cytoglobin regulates ligand binding. FEBS Lett..

[cit46] Romberg R. W., Kassner R. J. (1979). Nitric oxide and carbon monoxide equilibria of horse myoglobin and (N-methylimidazole)protoheme. Evidence for steric interaction with the distal residues. Biochemistry.

[cit47] Kruglik S. G., Lambry J. C., Cianetti S., Martin J. L., Eady R. R., Andrew C. R., Negrerie M. (2007). Molecular basis for nitric oxide dynamics and affinity with Alcaligenes xylosoxidans cytochrome c. J. Biol. Chem..

[cit48] Liebl U., Lambry J. C., Vos M. H. (2013). Primary processes in heme-based sensor proteins. Biochim. Biophys. Acta.

[cit49] Lobato L., Bouzhir-Sima L., Yamashita T., Wilson M. T., Vos M. H., Liebl U. (2014). Dynamics of the heme-binding bacterial gas-sensing dissimilative nitrate respiration regulator (DNR) and activation barriers for ligand binding and escape. J. Biol. Chem..

[cit50] Corti P., Xue J., Tejero J., Wajih N., Sun M., Stolz D. B., Tsang M., Kim-Shapiro D. B., Gladwin M. T. (2016). Globin X is a six-coordinate globin that reduces nitrite to nitric oxide in fish red blood cells. Proc. Natl. Acad. Sci. U. S. A..

[cit51] Gladwin M. T., Kim-Shapiro D. B. (2008). The functional nitrite reductase activity of the heme-globins. Blood.

[cit52] Jensen F. B. (2009). The role of nitrite in nitric oxide homeostasis: a comparative perspective. Biochim. Biophys. Acta.

[cit53] Shiva S., Huang Z., Grubina R., Sun J., Ringwood L. A., MacArthur P. H., Xu X., Murphy E., Darley-Usmar V. M., Gladwin M. T. (2007). Deoxymyoglobin is a nitrite reductase that generates nitric oxide and regulates mitochondrial respiration. Circ. Res..

[cit54] Tiso M., Tejero J., Basu S., Azarov I., Wang X., Simplaceanu V., Frizzell S., Jayaraman T., Geary L., Shapiro C., Ho C., Shiva S., Kim-Shapiro D. B., Gladwin M. T. (2011). Human neuroglobin functions as a redox-regulated nitrite reductase. J. Biol. Chem..

[cit55] Dewilde S., Kiger L., Burmester T., Hankeln T., Baudin-Creuza V., Aerts T., Marden M. C., Caubergs R., Moens L. (2001). Biochemical characterization and ligand binding properties of neuroglobin, a novel member of the globin family. J. Biol. Chem..

[cit56] Rohlfs R. J., Olson J. S., Gibson Q. H. (1988). A comparison of the geminate recombination kinetics of several monomeric heme proteins. J. Biol. Chem..

[cit57] Free M. J., Schluntz G. A., Jaffe R. A. (1976). Respiratory gas tensions in tissues and fluids of the male rat reproductive tract. Biol. Reprod..

[cit58] Massie E. D., Gomes W. R., VanDemark N. L. (1969). Oxygen tension in testes of normal and cryptorchid rats. J. Reprod. Fertil..

[cit59] Reyes J. G., Farias J. G., Henriquez-Olavarrieta S., Madrid E., Parraga M., Zepeda A. B., Moreno R. D. (2012). The hypoxic testicle: physiology and pathophysiology. Oxid. Med. Cell. Longevity.

[cit60] Corti P., Ieraci M., Tejero J. (2016). Characterization of zebrafish neuroglobin and cytoglobins 1 and 2: Zebrafish cytoglobins provide insights into the transition from six-coordinate to five-coordinate globins. Nitric Oxide.

[cit61] Balercia G., Moretti S., Vignini A., Magagnini M., Mantero F., Boscaro M., Ricciardo-Lamonica G., Mazzanti L. (2004). Role of nitric oxide concentrations
on human sperm motility. J. Androl..

[cit62] Dutta S., Sengupta P. (2022). The Role of Nitric Oxide on Male and Female Reproduction. Malays. J. Med. Sci..

[cit63] Muller U., Bicker G. (1994). Calcium-activated release of nitric oxide and cellular distribution of nitric oxide-synthesizing neurons in the nervous system of the locust. J. Neurosci..

[cit64] Van Hove C. E., Van der Donckt C., Herman A. G., Bult H., Fransen P. (2009). Vasodilator efficacy of nitric oxide depends on mechanisms of intracellular calcium mobilization in mouse aortic smooth muscle cells. Br. J. Pharmacol..

[cit65] Charles A. (1999). Nitric oxide pumps up calcium signalling. Nat. Cell Biol..

[cit66] Lee J. H., Kim H., Kim D. H., Gye M. C. (2006). Effects of calcium channel blockers on the spermatogenesis and gene expression in peripubertal mouse testis. Arch. Androl..

