# Supplementary material for: The circularly permuted globin domain of androglobin exhibits atypical heme stabilization and nitric oxide interaction
Source: Chem Sci. 2024 Mar 21;15(18):6738–51. doi: 10.1039/d4sc00953c (PMC11077535; doi:10.1039/d4sc00953c)
Supplement: SC-015-D4SC00953C-s001 [file SC-015-D4SC00953C-s001.pdf]

Electronic Supplementary Material (ESI) for Chemical Science.
This journal is © The Royal Society of Chemistry 2024

Electronic Supporting Information

The circularly permuted globin domain of Androglobin exhibits atypical heme stabilization and nitric oxide interaction

Brandon J. Reeder*[a], Giuseppe Deganutti[a,b], John Ukeri[a], Silvia Atanasio[a], Dimitri A. Svistunenko[a], Christopher Ronchetti[a], Juan Carlos Mobarec[a], Marten H. Vos[c], Elizabeth Welbourn[a], Jeffrey Asaju[a], Michael T. Wilson[a], and Christopher A Reynolds*[a,b]

[a] School of Life Sciences, University of Essex, Wivenhoe Park, Colchester, Essex, CO4 3SQ, UK;

[b] Centre for Life Sciences (CHLS), Alison Gingell Building, Coventry, CV1 5FB, UK;

[c] LOB, CNRS, INSERM, École Polytechnique, Institut Polytechnique de Paris, 91128 Palaiseau, France.

Email: reedb@essex.ac.uk (biochemical), and ad5291@coventry.ac.uk (modelling)

Table of Contents

S1. Materials S2

S2. The androglobin alignment........ S2

S3. Modelling the androglobin structure........ S2

S4. Molecular dynamics studies of complex 1 and complex 2........ S2

S5. Androglobin globin domain expression and purification........ S3

S6. Determination of androglobin globin domain absorption coefficient........ S3

S7. Size exclusion, SDS PAGE and western blot analysis. S3

S8. Determination of androglobin globin domain disulfide........ S4

S. Electron Paramagnetic Resonance Spectroscopy........ S4

S10. Stopped flow spectroscopy........ S4

S11. Femtosecond laser flash photolysis........ S4

S12. Supplementary Figures and Tables........ S5

S13. References........ S10

S1. Materials

Horse heart Mb, sodium oleate, 4,4'-dithiodipyridine, tris(2-carboxyethyl)phosphine (TCEP) and all buffer materials were purchased from Sigma-Aldrich (Merck), Poole, UK. Isopropyl β-D-thiogalactopyranoside (IPTG) and 5-aminolaevulinic acid were from Molekula, Gillingham, UK. Proli-Nonoate was purchased from Cayman Chemicals via Cambridge Bioscience UK. Carbon monoxide was purchased from BOC, UK.

S2. The androglobin alignment

The alignment was carried out using a novel multi-template profile alignment based on multiple pairwise alignments.[1] The alignments for Adgb, alpha-Hb, beta-Hb, Cygb, Mb, and Ngb were determined by (i) collecting sequences from the NCBI (https://www.ncbi.nlm.nih.gov/) non-redundant database using BLASTp, [2] (ii) sifting the BLASTp output for keywords, e.g. Cygb, so that there was no cross-contamination by other globins, (iii) eliminating 'predicted' (i.e. mRNA-based) sequences so only high quality sequences were included, (iv) alignment using Clustal Omega [3] and (v) manual editing using Jalview.[4] Sequences more than 90 % similar to other sequences were removed. The structural alignment of alpha-Hb, beta-Hb, Cygb, Mb and Ngb (PDB codes: 1BZ1, 1DXT, 1UT0, 3RGK, and 4MPM respectively) was determined using the align module of Modeller;[5] since these proteins align to give a well-defined sequence alignment, the alignment of Adgb to one of these 5 was essentially equivalent to alignment to the others and this forms a key aspect of the multi-template alignment. Profiles for the main helices A-B, E-H were generated for each protein with a central region of 13 residues and a flank of 25 residues on either side. For each helix, the profile alignment between Adgb and each of alpha-Hb, beta-Hb, Cygb, Mb, and Ngb was carried out by doing all possible pairwise alignments and counting the votes for the top alignment, as described elsewhere[1, 6, 7]. The averaged votes were scaled between 0 and 1 and reported in Fig. 1 (left hand side) for each helix and for each protein alpha-Hb, beta-Hb, etc. A consensus alignment was determined by multiplying the 5 sets of votes together, as shown in Fig. 1 (right-hand side). Control alignments were also carried out for all possible pairs of alpha-hemoglobin, beta-hemoglobin, cytoglobin, myoglobin, and neuroglobin; these were in agreement with the structural alignment (results not shown). The alignment is shown in Fig. 2, along with the 2012 alignment 1 (for the key sections where it differs from the one reported here).

S3. Modelling the androglobin structure

Two initial models of Adgb-GD were generated using Modeller.[8] One was built on the assumption that Adgb was not cyclically permuted but rather had the same topology as the other Hb molecules (i.e. assuming residues for helix A-H are contiguous in the sequences). The second was built taking into account the unique circularly-permuted Adgb topology, but omitting the IQ domain, residues 891-933. The alignment was taken from Fig. 2; the Protein Data Bank (PDB) codes for the structural templates were 1BZ1, 1DXt, 1UT0, 3RGK, and 4MPM. Both structures were built with and without the Cys787 and Cys978 disulfide bond. In each case, 2000 Modeller structures were generated and the model with the lowest DOPE score was selected for MD studies. Initial 0.5 µs MD simulations on these models suggested that the structure was stable (results not shown) and on this basis the expression studies were initiated.

A region of the IQ calmodulin-binding motif (IQ refers to the first 2 amino acids of the motif) was predicted to be helical by both PsiPred[9] and Jpred.[10] Consequently, this region was docked to the above permuted Adgb-GD model using cluspro, [11] giving two main poses, one on either side of the heme; from these structures two alternative templates were generated for inclusion with the other structural templates, (i) Ala904-Ala928 docked to helices G and H (complex 1) and (ii) Ala904-Ala928 docked to helices E and F (complex 2). Residues Glu905 – Ala928 were restrained to be helical within Modeller. In each case, 2000 structures were generated and the two models with the lowest DOPE score was selected for further MD studies (the DOPE score indicated that complex 2 was more stable than complex 1).

S4. Molecular dynamics studies of complex 1 and complex 2

The AlphaFold 2 [12, 13] model of the human androglobin, which does not present the heme bound to Adgb-GD, was retrieved at https://alphafold.ebi.ac.uk/entry/Q8N7X0. Complex 1, complex 2, and the Adgb-GD of AlphaFold 2 model (residues Glu785 to Lys985) were prepared for molecular dynamics (MD) simulations with the CHARMM36 force field.[14] An in-house procedure that combines HTMD[15] and Tcl (Tool Command Language) scripts allowed the addition of hydrogen atoms by means of the pdb2pqr [16] and propka[17] software (considering a simulated pH of 7.0); the protonation of titratable side chains was checked by visual inspection. TIP3P water molecules[18] were added to the simulation box by means of the VMD Solvate plugin 1.5 (Solvate Plugin, Version 1.5. at http://www.ks.uiuc.edu/Research/vmd/plugins/solvate/) with a padding of 15 Å. Overall charge neutrality was finally reached by adding Na^+/Cl^- counter ions (final ionic strength of 0.150 M), using the VMD Autoionize plugin 1.3 (Autoionize Plugin, Version 1.3 at http://www.ks.uiuc.edu/Research/vmd/plugins/autoionize/). The proximal histidine (His824) was patched to the heme iron atom according to the CHARMM36 PHEM patch.

The MD engine ACEMD[19] was employed for both the equilibration and productive simulations. Equilibration was achieved in two steps: after 500 cycles of conjugate-gradient minimization, the systems were simulated for 4 ns employing an integration time step of 2 fs in the isothermal-isobaric conditions (NPT), using the Berendsen barostat [20] (target pressure 1 atm) and the Langevin thermostat [21] (target temperature 300 K) with a low damping of 1 ps^{-1}. Positional restraints of 1 kcal mol^{-1} $Å^{-2}$ on protein and heme atoms were gradually reduced to zero.

MD trajectories were computed with an integration time step of 4 fs in the canonical ensemble (NVT) at 300 K, using a thermostat damping of 0.1 ps^{-1} and the M-SHAKE algorithm [22] to constrain the bond lengths involving hydrogen atoms. The cut-off distance for electrostatic interactions was set at 9 Å, with a switching function applied beyond 7.5 Å. Long range Coulomb interactions were handled using the particle mesh Ewald summation method (PME)[23] by setting the mesh spacing to 1.0 Å. Complex 1 and 2 were simulated in triplicate for 500 ns each replica, while a single 2 µs replica was performed on the AlphaFold 2 model.

Root Mean Square Deviation (RMSD) and Root Mean Square Fluctuation (RMSF) were computed using VMD.[24] Distances between cysteine residues were computed using MDTraj.[25]

S5. Androglobin globin domain expression and purification

The gene for the Adgb-GD, codon optimized for *E. coli* expression, was synthesized by Epoch Life Sciences Inc, Missouri City, USA and incorporated into expression vector pET28a (Merck) using NcoI/XhoI restriction sites. The vector was transformed into BL21 DE3 cells (Sigma-Aldrich, UK) by heat shock method (42 ºC, 90 s). Protein was expressed in shaking flasks containing 1.4 L Luria-Bertani media with kanamycin sulfate (50 µg ml^{-1}), shaken at 180 rpm, 37 ºC. When optical density was ~1.0 at 600 nm, protein expression was initiated by addition of 500 µM IPTG to activate the T7 promotor system for protein expression. Ferric citrate (50 µM) and 5-aminolaevulinic acid (250 µM) were added to the media to augment heme synthesis. Carbon monoxide gas was bubbled through the solution for 30 s to stabilize the protein following expression and to prevent autoxidation and associated radical production that can lead to oxidative protein and heme modifications. The flasks were sealed using a rubber bung and incubated for a further 18 h, shaken at 80 rpm, 32 ºC. Cells were isolated by centrifugation (8000 g, 20 min), and cells were resuspended in water and frozen at -20ºC. Defrosted cells were lysed using an Avestin Emulsiflex C3 homogenizer at 15000-20000 psi, 2 passes. Following centrifugation (30000 g, 20 min, 4ºC) supernatant was separated and buffer added to a final concentration of 20 mM sodium phosphate, 500 mM sodium chloride, 0.25 % SDS and 20 mM imidazole, pH 7.4. The cell pellet contains significant amounts of Adgb, so the pellet was resuspended in 20 mM sodium phosphate, 500 mM sodium chloride, 1 % SDS and 20 mM imidazole, pH 7.4. The suspension was centrifuged (30000 g, 20 min, 4ºC) and the supernatant diluted to 0.25 % SDS with 20 mM sodium phosphate, 500 mM sodium chloride and 20 mM imidazole, pH 7.4. The two supernatant solutions were pooled.

The His-tagged protein was purified using a GE Healthcare immobilized metal (nickel) affinity column (5 ml), washed with phosphate buffer, 500 mM sodium chloride, 0.25 % SDS with 20 mM imidazole and eluted with phosphate buffer, 500 mM sodium chloride with 500 mM imidazole. Imidazole was removed by dialysis (1 mM sodium tetraborate, pH 9.5, 3 changes) and the His-tag was cleaved through incubation with bovine thrombin (Sigma–Aldrich; 10 units/mg of protein) at room temperature (22ºC) overnight with gentle mixing in 20 mM Tris pH 8.4, 150 mM NaCl, 2.5 mM $CaCl_2$. Tag-free protein was purified using the nickel-affinity column (20 mM sodium phosphate, 500 mM sodium chloride, 0.25 % SDS and 20 mM imidazole, pH 7.4), dialysed and concentrated using a Whatman 3 kDa spin filter. SDS can be replaced with deoxycholate, tween 20 or triton-X100 to increase yields of apoprotein, however, highest yields were achieved using SDS.

During purification some loss of heme was experienced and required heme reconstitution. A molar ratio of 1:0.95 protein:hemin (from a 5 mM hemin solution dissolved in 10 mM sodium hydroxide) was added based on a calculated 280 nm extinction coefficient of 32.5 mM^{-1} cm^{-1} for the apo protein and left overnight at 4 °C followed by gel filtration to remove unbound heme (Sephadex G25, 5x1 cm column, 10 mM sodium phosphate pH 7.4). The protein was stored as the ferric form in sodium phosphate or borate buffer (~1 mM) and can be frozen and defrosted several times without stability issues.

In samples requiring reduction of the disulfide bond, this was achieved by addition of 1 mM tris(2-carboxyethyl)phosphine (TCEP) to ~100 µM Adgb-GD and incubated at 25 °C for one hour. TCEP was removed by size exclusion chromatography (Sephadex G25, 5 x 1.45 cm column).

S6. Determination of androglobin globin domain absorption coefficient

Unknown concentrations of ferric Adgb-GD, along with a known concentration of Mb (calculated using the ferrous protein, molar absorption coefficient ($ε_{435nm}$ = 121 mM^{-1} cm^{-1})[26] had their optical properties recorded (ferric, deoxyferrous, ferrous-NO, -CO) before they were subjected to reverse-phase HPLC using an Agilent 1100 HPLC fitted with a diode array spectrophotometer. The column used was a Zorbax Stablebond 300C3 250 mm × 4.6 mm fitted with a 12 mm × 4.6 mm guard column, with a water/TFA – acetonitrile/TFA gradient as previously described.[27, 28] The concentration of heme in the unknown Adgb-GD sample was calculated from the integrated area under the peak (∼15 min elution time) and compared to

the integral of the heme peak of the known concentration of Mb. This was repeated on three independent samples. The ε_{412nm} of ferric Adgb-GD pH 7.4 was calculated to be 106 mM^{-1} cm^{-1} and was used for concentration determination in all experiments.

S7. Size exclusion, SDS PAGE and western blot analysis.

Protein oligomerisation was measure by size exclusion analysis using an Agilent 1290 UHPLC fitted with a diode array spectrophotometer. Column used as an Agilent Advance BioSEC 130A 2.7 µm column, 7.8x300 mm with isocratic elution using 100 mM sodium phosphate buffer with or without 150 mM sodium chloride. Molecular weight calibration was performed using a mixture of 2 mg/ml bovine serum albumin (monomer and dimer), equine myoglobin, equine cytochrome c and ferricyanide. SDS PAGE analyses were performed using 4 % gels. Each analysis was carried out with 1 µg sample loading per well. Electrophoresis was carried out for 35 min at 200 V. Gels were stained using InstantBlue™ (Expedeon) and imaged using an ImageQuant™ LAS4010 system (GE Healthcare) and analyzed using GelAnalyzer vs 23.11. Protein ladder was Thermo Scientific PageRuler™ prestained protein ladder.

Western blot was measured as follows: Polyvinylidene fluoride (PVDF) membranes were pre-wetted in 100% methanol for 1 minute. SDS-PAGE gel and PVDF membranes were soaked in chilled transfer buffer (25 mM Tris, 192 mM glycine, 0.1% SDS and 20% methanol) for 15 minutes. Proteins were transferred to the PVDF membrane using a BioRad Mini Trans-Blot cell according to manufacturer's instructions. Following transfer, the membrane was blocked overnight in Milk (10% skimmed milk powdered (Marvel) and 0.1% tween-20) in phosphate buffered saline (PBS). 6X His-Tag monoclonal antibody (His.H8), HRP from Thermofisher, UK was diluted 1:5000 in 0.1% tween-20 PBS solution and incubated with the membrane for one hour. The membrane was washed five times with 0.1% Triton X-100 PBS solution. To generate a chemiluminescent signal, SuperSignal West Pico PLUS chemiluminescent substrate was used according to manufacturer's instructions. The visualization of the blots was performed using Fusion Fx Vilber Lourmat imaging system

S8. Determination of androglobin globin domain disulfide

The extent of free cysteine sulfhydryls was determined by the dithiodipyridine assay as described previously.[29] Protein (10 µM) was titrated with 4,4'-dithiodipyridine and the absorbance monitored at 324 nm. This was repeated following protein disulfide reduction by TCEP. The titration curve was fitted to a fractional saturation curve using the least-squares method by Microsoft Excel solver program as previously described.[30]

S9. Electron Paramagnetic Resonance Spectroscopy

Adgb aliquots (80 µM, 250 µl) were placed in Wilmad SQ EPR tubes (Wilmad Glass, Buena, NJ). Nitric Oxide was added immediately prior to freezing by addition of Proli-Nonoate (1 mM, giving 2 mM NO) in 10 mM sodium hydroxide. Tubes were flash-frozen in dry-ice-cooled methanol and frozen samples were transferred to liquid nitrogen (77 K). A Bruker EMX and a Bruker E500 EPR spectrometers (both Xband) were used, each combined with Oxford Instruments liquid helium systems, to measure low temperature continuous wave (CW) EPR spectra. The modulation frequency was 100 kHz. The EPR spectra of the blank samples (frozen water) were subtracted from the EPR spectra of the protein samples using WinEPR v. 2.22 (Bruker Analytik, GmbH) to eliminate the baseline caused by the walls of the resonator, quartz insert or quartz EPR tube.

S10. Stopped flow spectroscopy

Stopped-flow absorbance spectroscopy was used to measure the kinetics of change in heme iron co-ordination using an Applied Photophysics SX20 stopped-flow spectrometer fitted with a diode array spectrometer with time resolution of 1.25 ms. For the measurement of NO binding, buffer (100 mM sodium phosphate buffer pH 7.4) was degassed using a glass tonometer connected to a supply of argon gas and a vacuum pump via a custom build glass tap system to allow repeated degassing cycles. The degassed buffer was transferred anaerobically to a 10 ml glass syringe. Proli-NONOate was prepared in 25 mM sodium hydroxide to a concentration of 40 mM and then purged with argon gas. Proli-NONOate was transferred using a glass Hamilton syringe to the degassed buffer (12.5–200 µM), under these conditions the Proli-NONOate degrades to generate 1.8 of NO within a few milliseconds (measured as 1.8 molecules of NO per proliNONOate). [31] The NO resulting from this degradation of proli-NONOate was rapidly mixed in a 1:1 ratio with 10 µM Adgb (5 µM final concentration) in 100 mM sodium phosphate buffer (pH 7.4) in degassed buffer with minimal dithionite added to keep the protein reduced and oxygen free. For the nitrite reductase activity Adgb (10 µM, 5 µM final) was rapidly mixed in a 1:1 ratio with sodium nitrite (2-40 mM, 1-20 mM final). Both Adgb and nitrite solutions were oxygen free by use of excess sodium dithionite.

S11. Femtosecond laser flash photolysis

Ultrafast broad-band transient absorption experiments at a 500 Hz repetition rate, were performed as previously described, [32] using pump pulses centered at 570 nm and white-light continuum probe pulses. The sample was continuously rastered by a Lissajous scanner. Global analysis of the data in terms of multi-exponential functions was performed using Glotaran.

S12. Supplementary Figures and Tables

Table S1. The mean percentage identity for the pairwise alignments for each helix-helix profile alignment between androglobin and the other hemoglobins.

	**A helix**	**B helix**	**E helix**	**F helix**	**G helix**	**H helix**
**α-Hb (alpha)**	15.3	16.2	15.1	27.6	13.7	10.3
**β-Hb (beta)**	15.2	11.1	12.1	21.5	14.2	9.7
**Cygb (cyto)**	16.6	23.7	19.8	21.5	13.9	11.2
**Mb (myo)**	21.1	18.7	22.2	25.8	8.9	11.3
**Ngb (Neuro)**	15.1	15.5	9.1	25.0	13.6	7.8

Table S2. Frequency analysis of amino acid CD1 assignment. Human androglobin sequence was aligned with 771 androglobin sequences. Left: amino acid frequency as previously published based on residue 769. Right: revised amino acid frequency based on residue 975.

**CD1 Assignment: Residue 769**		**CD1 Assignment: Residue 975**	
Amino Acid	Frequency (%)	Amino Acid	Frequency (%)
Phe	75.38	Tyr	81.84
Cys	22.02	Phe	16.78
Other	2.60	Other	1.38

Table S3. Wavelength maxima and extinction coefficients of Adgb-GD and other globins. For Adgb-GD, buffer was 100 mM sodium phosphate buffer pH 7.4.

Protein	Oxidation and ligation state	Soret peak, nm ($mM^{-1}cm^{-1}$)	Beta peak, nm ($mM^{-1}cm^{-1}$)	Alpha peak, nm ($mM^{-1}cm^{-1}$)	Ref
**Human Adgb-GD**	Ferric	412 (106)	534 (12.8)	566 (10.1)	This work
	Deoxyferrous	426 (108)	531 (12.2)	560 (17.8)	This work
	Ferrous-CO	422 (180)	540 (16.0)	560 (15.0)	This work
	Ferrous-NO	395 (95)	540 (14.4)	559 (14.1)	This work
**Equine Hb**	Ferric (H_2O)	405 (179)	500 (10.0)	631 (4.4)	26
	Ferric (OH^-)	410 (120)	540 (11.0)	575 (9.2)	26
**Human Hb**	Deoxyferrous	430 (133)	555 (12.5)		26
	Deoxyferrous-CO	419 (191)	540 (13.4)	569 (13.4)	26
	Deoxyferrous-NO		545 (12.6)	575 (13.0)	26
**Equine Mb**	Ferric (H_2O)	408 (188)	502 (10.2)	630 (3.9)	26
	Ferric (OH^-)	411 (119)	539 (8.8)	585 (7.8)	26
	Deoxyferrous	435 (121)	560 (13.8)		26
	Deoxyferrous-CO	424 (207)	540 (15.4)	579 (13.9)	26
	Deoxyferrous-NO		543 (11.6)	575 (10.5)	26
**Human Ngb**	Ferric	414 (129)			33
	Deoxyferrous		528 (16.2)	558 (28.9)	34
	Deoxyferrous-CO	420 (119)	538 (11.5)	561 (11.2)	34, 35
	Deoxyferrous-NO	416 (118)	550 (11.1)		36
**Human Cygb**	Ferric	416	535	565	37
	Deoxyferrous	428 (165)			27
	Deoxyferrous-CO		542	570	37

Table S4. Binding constants and rates of Adgb-GD. *Calculated from initial slope. NDB: does not bind. K_D calculated from k_{off}/k_{on} except for cyanide binding.

	k_{on} ($M^{-1}s^{-1}$)	k_{off} (s^{-1})	K_D (M)
**Nitric Oxide (to ferrous)**	≥1x10^{7} (fast)<br>≥1 x10^{6} (slow)	1.60 x10^{-4} ± 0.07 x10^{-4}	≥1.6x10^{-11} (fast)_<br>≥1.6x10^{-10} (slow)
**Nitrite reductase**	25.1*	0.25 ± 0.03	8.42 x10^{-3}± 1.96 x10^{-3}
**Nitrite reductase (TCEP reduced disulfide)**	7.2*	0.037 ± 0.004	3.26 x10^{-3} ± 1.12 x10^{-3}
**CO**	≥3x10^{6} (fast)<br>≥3 x10^{5} (slow)	0.31 ± 0.05	≥9x10^{-8} (fast)_<br>≥9x10^{-7} (slow)
**O_2**	1.3 x10^{-5}	-	-
**Cyanide**	-	-	16.35 x10^{-3} ± 5.9 x10^{-4}
**Azide**	NBD	NBD	>>5 x10^{-2}

Table S5. Rate constants k_i ($x10^{10}$ s^{-1}) for geminate recombination of NO to ferrous Androglobin globin domain, other globins (all forming 6-coordinate ferrous NO complexes), and other heme proteins forming 5-coordinate ferrous NO complexes. All are human complexes unless otherwise stated. The relative amplitudes of the recombination phases (and of the constant phase (c), which corresponds to the fraction that escapes the protein) are given between brackets. DNR represents Dissimilatory Nitrate Respiration regulator and sGc represents soluble Guanylyl cyclase

**Protein**	**k_1**	**k_2**	**k_3**	**c**	**Reference**
**Adgb-GD**	19 *(0.85)*	5.0 *(0.14)*	-	*0.01*	This work
**Mb**	4.08 *(0.44)*	0.71 *(0.49)*	-	*0.08*	[43]
**Mb (equine)**	20 *(0.53)*	2.0 *(0.22)*	0.20 *(0.21)*	*0.04*	[44]
**Ngb**	20 *(0.62)*	4.8 *(0.28)*	0.38 *(0.06)*	*0.04*	[45]
**sGc (bovine)**	13 *(0.97)*	-	-	*0.03*	[46]
**Cyt *c'* *(A. xylosoxidans)***	14 *(1.00)*	-	-	*0.00*	[47]
**DNR *(P. aeruginosa)***	14 *(1.0)*			*0.0*	[32]

Table S6. Nitrite reductase activity of various members of the hemoglobin family. All are human globins unless otherwise stated.

Protein	k_{NiR} ($M^{-1}s^{-1}$)	Temperature	Reference
Cytoglobin (intramolecular disulfide)	32.3	25	[38]
Zebrafish Cytoglobin 1	14.6<br>28.6	25<br>37	[39]
Zebrafish Globin X	26.7	37	[40]
Androglobin Globin Domain	25.1	25	This work
Hemoglobin R state	6.0	25	[41]
Equine Myoglobin	2.7	25	[38]
Cytoglobin (reduced SH)	0.63	25	[38]
Zebrafish Cytoglobin 2	0.31	25	[39]
Cytoglobin (intermolecular disulfide)	0.26	25	[38]
Hemoglobin T state	0.12	25	[41]
Neuroglobin (intramolecular disulfide)	0.12	25	[42]
Neuroglobin (reduced SH)	0.062	25	[42]

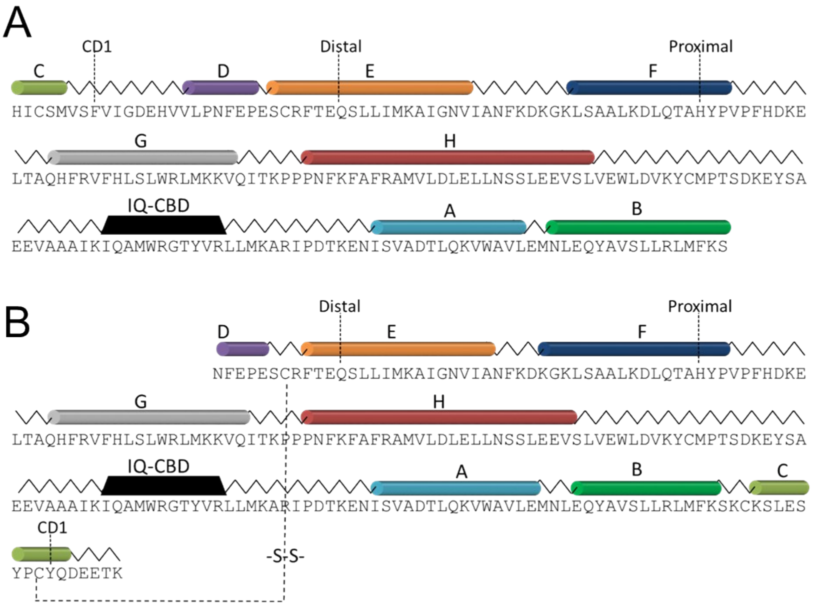

C

MGSSHHHHHHSSGLVPRGSHNFEPESCRFTEQSLLIMKAIGNVIANFKDK 50
GKLSAALKDLQTAHYPVPFHDKELTAQHFRVFHLSLWRLMKKVQITKPPP 100
NFKFAFRAMVLDLELLNSSLEEVSLVEWLDVKYCMPTSDKEYSAEEVAAA 150
IKIQAMWRGTYVRLLMKARIPDTKENISVADTLQKVWAVLEMNLEQYAVS 200
LLRLMFKS**KCKSLESYPCYQDEETK***

Figure S1. Androglobin heme-binding globin domain structure alignments. (A), Original alignment of alpha helical sequences to the classical 8-helix globin fold and IQ calmodulin binding domain (IQ-CDB) as reported previously (residues 763-968) [48]. Distal glutamine and proximal histidine residues are highlighted in the E and F helical sections, as well as heme-stabilizing CD1 phenylalanine at the N terminal region. (B), Alternative sequence alignment with similar/identical A-B and E-H helices but differing C-D alignment (residues 781-985. This revised alignment places the CD1 residue on the C terminal region as a tyrosine. In addition, this predicts the potential for the formation of a disulfide bond (-S-S-) in the CD 'loop' similar in position to that observed in human Ngb. The recombinant expression of the revised sequence allowed stable Adgb-GD protein to be expressed. (C), Protein sequence as expressed including N-terminal His-tag sequence and linker highlighted in bold italics, thrombin cleavage site indicated by arrow and redefined C helix region at the C terminus in bold.

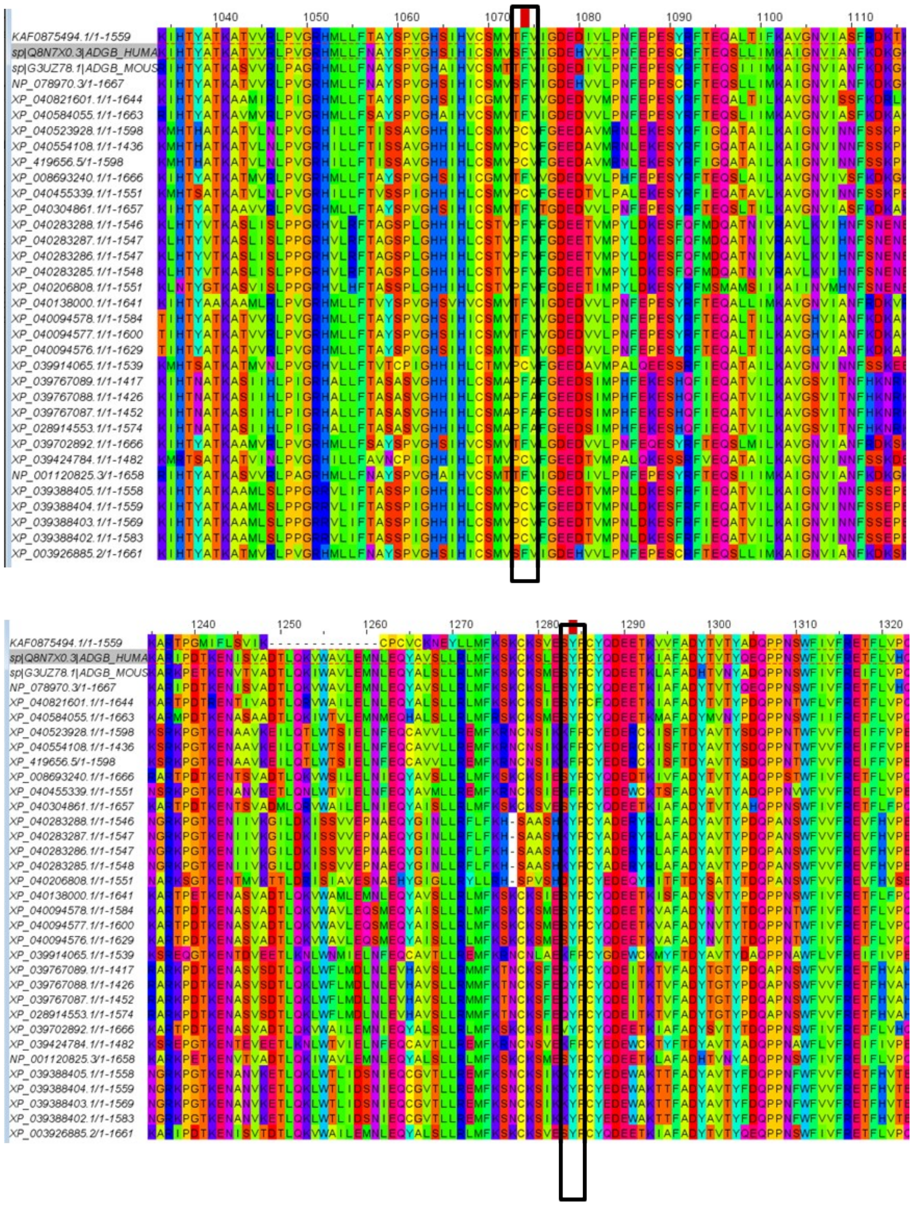

Figure S2. Multiple sequence alignments for androglobin globin domain showing the conservation at the previously proposed CD1 Phe/Cys (Phe770 for human Adgb) [30] or CD1 Tyr/Phe.

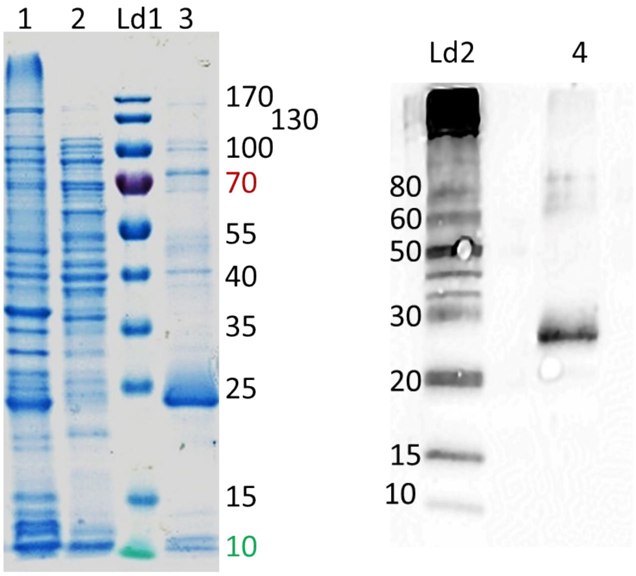

Figure S3. Left, SDS-PAGE of purification of Adgb-GD. Lane 1, cell pellet following lysis and centrifugation but before re-solubilization using 1% SDS. Lane 2, cell lysate following lysis and centrifugation. Ld1, Ladder (Thermo Scientific PageRuler™ prestained protein ladder). Lane 3, protein following purification using IMAC column (GE healthcare HisTrap HP 5ml). Right, western blot. Ld2, Histag ladder (Thermo Fisher BenchMark His-tagged Protein Standard). Lane 4, purified protein.

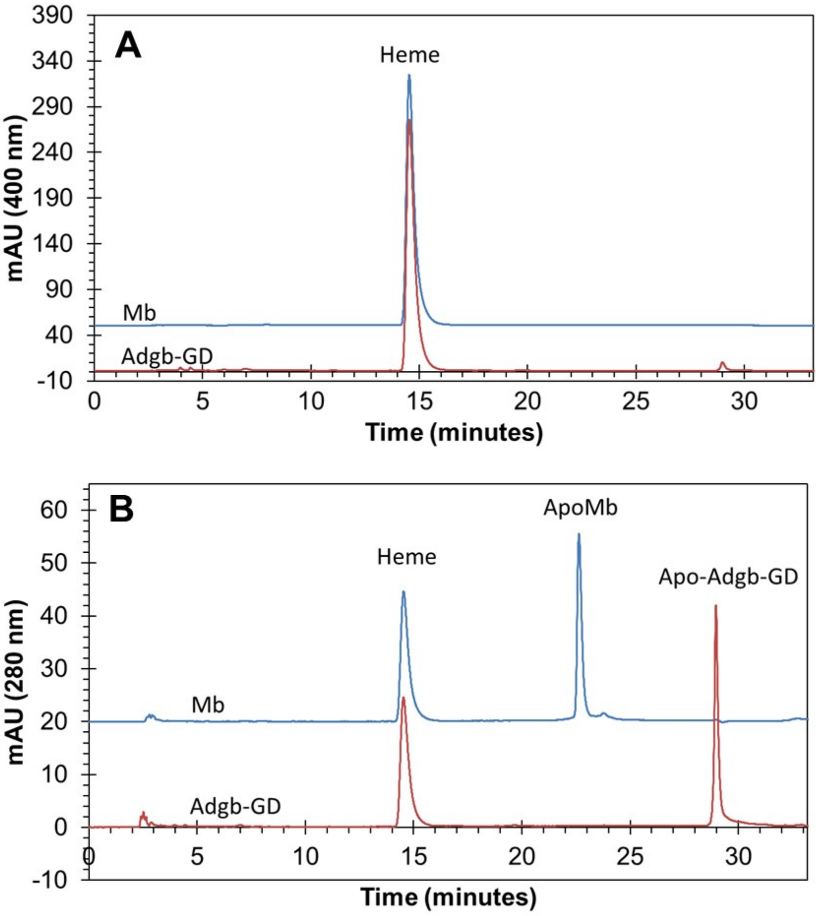

Figure S4. Reverse phase HPLC analysis of globin heme content of Androglobin globin domain using myoglobin as a control. Equine myoglobin (Mb), premeasured as 50 µM per heme content by optical spectroscopy, was analyzed by reverse phase HPLC as described in the methods. This was repeated with three independent samples. The area underneath the heme peak at 400 nm (A) was determined using the Agilent Chemstation software (version B.01.03). Androglobin globin domain (Adgb-GD) as analyzed and the area underneath the heme peak was measured and compared to the Mb samples to determine sample concentration. This was repeated three times and was then used to determine extinction coefficients from the optical spectra measured from the same samples. Apo-protein can be observed separated from the heme at 280 nm with no evidence of covalent linkage between the protein and heme (B). Traces are offset for clarity.

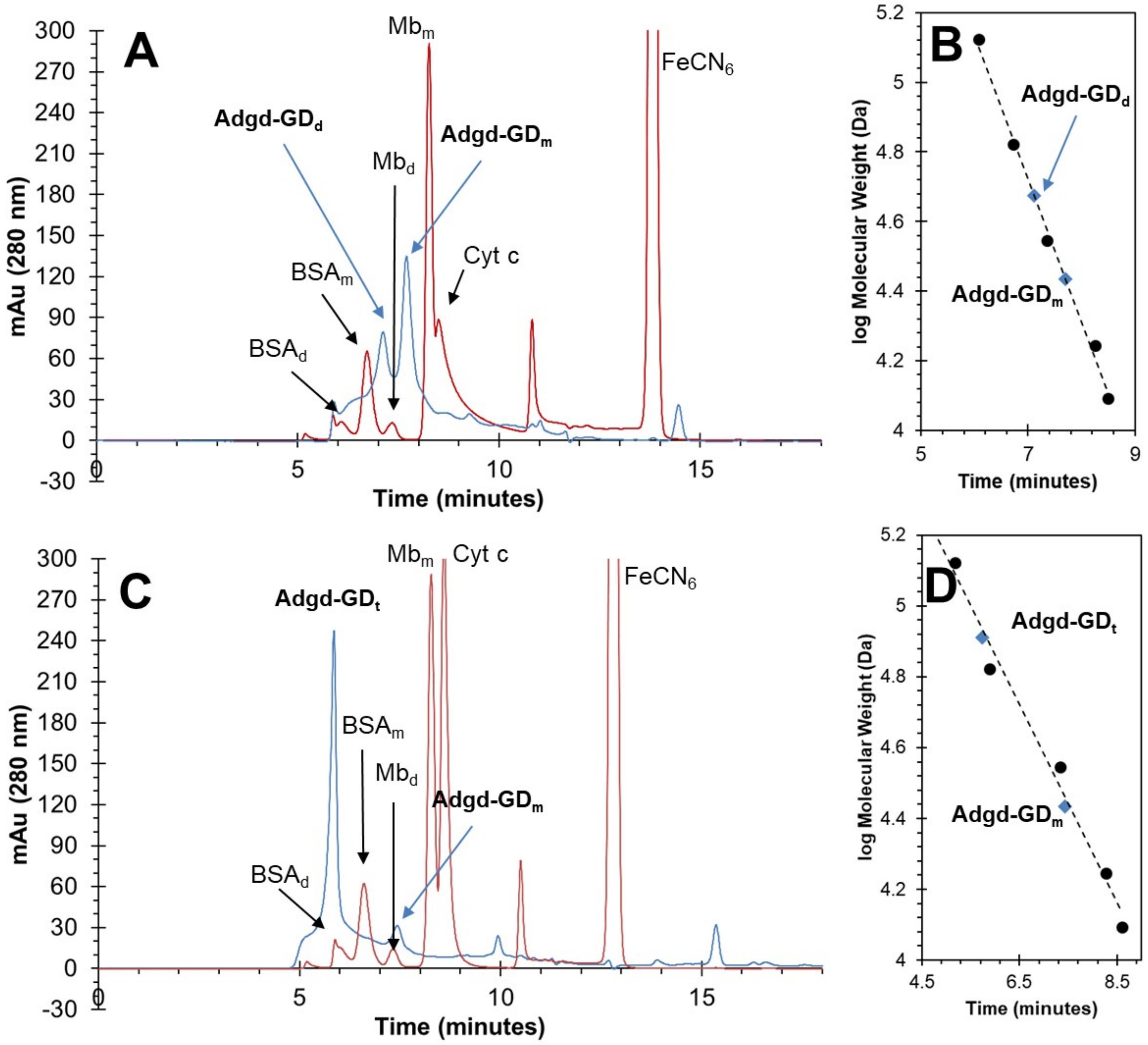

Figure S5. Size exclusion chromatography of Adgb-GD. (A) Adgb (blue line) in 0.1 M sodium phosphate buffer pH 7.4, 25 °C shows a mixture of monomer (Adgb-GD$_m$) and dimer (Adgb-GD$_d$). The column was calibrated for each condition using a mixture (red line, each 2 mg.ml^{-1}) of bovine albumin (containing dimer, BSA$_d$ and monomer BSA$_m$), equine myoglobin (containing dimer, Mb$_d$ and monomer Mb$_m$), equine cytochrome c (Cyt c) and ferricyanide (FeCN$_6$). (B) Calibration curve of mixture (black circles). Theoretical molecular weights of monomeric and dimeric Adgb-GD were plotted (blue diamonds) along the y axis plotted at the elution time (x axis). (C) Adgb (blue) as for (A) except buffer contained addition of 150mM NaCl. Here Adgb is primarily tetrameric. (D) Calibration curve of mixture (black circles). Theoretical molecular weights of monomeric and dimeric Adgb-GD were plotted (blue diamonds) along the y axis plotted at the elution time (x axis). All samples were analyzed using an Agilent 1290 UHPLC fitted with an AdvanceBio SEC 130Å 2.7 µm 7.8x300 mm column.

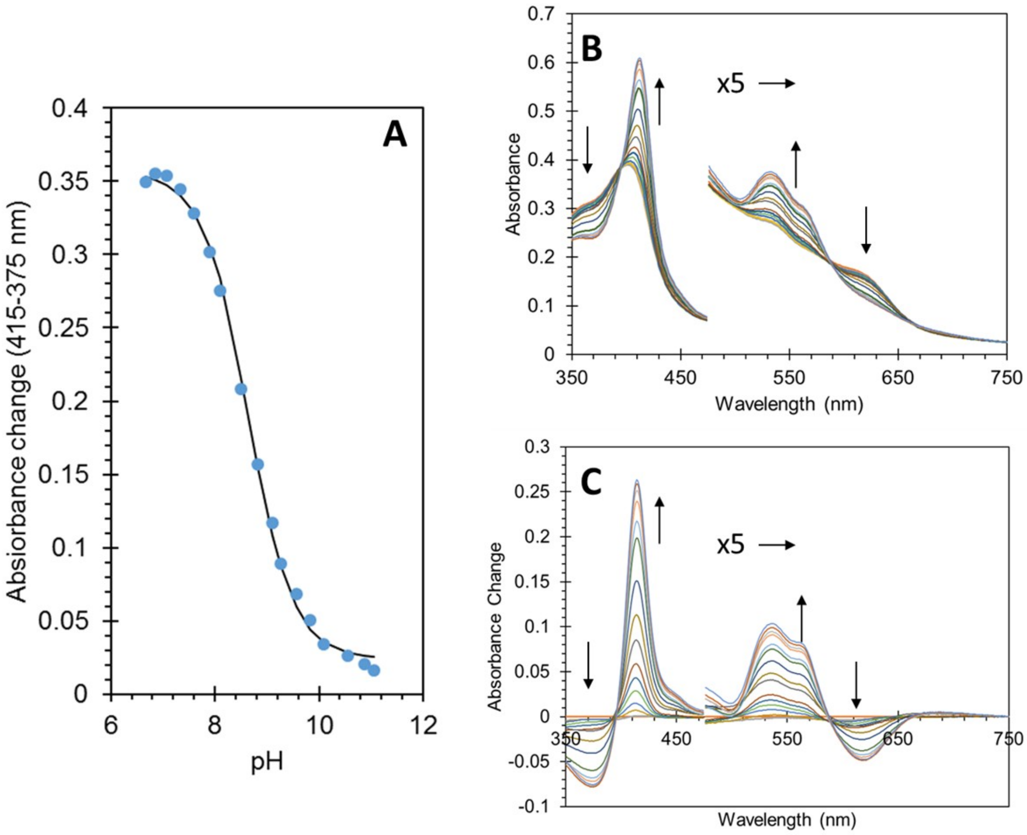

Figure S6. The acid–alkaline transition of ferric Adgb. Ferric Adgb-GD (5 µM) was adjusted to approx. pH 11 by addition of buffer (10 mM sodium phosphate and 10 mM sodium tetraborate). Acid (HCl 0.05–5 M) was titrated into the solution and the pH and optical spectra were recorded. There is a single acid–alkaline transition with pK of 8.64 ± 0.02 (A). Optical changes for absolute (B) and difference (C) spectra confirm a single transition from pentacoordinate at high pH and hexacoordinate at low pH. The vertical arrows depict the change in the spectra as pH is decreased from 11 to 6.5.

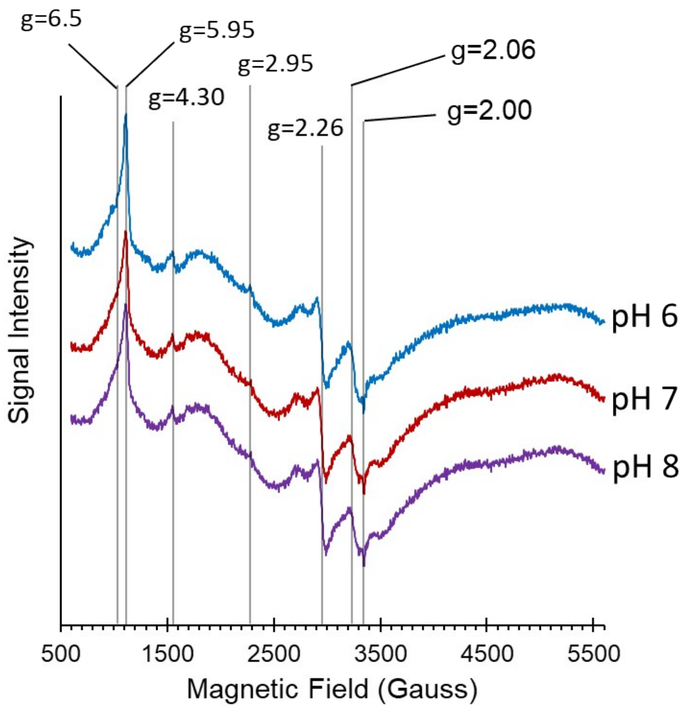

Figure S7. Androglobin globin domain heme iron EPR under reduced cysteine oxidation. EPR spectra of 80 µM ferric Adgb-GD domain following reduction of disulfide bond by TCEP (see Methods). As with the EPR data with oxidised cysteines (Fig 6A), the TCEP reduced protein exhibits both high spin and low spin Fe^{3+} EPR signals at various pH values. The spectra were recorded at 10 K, 3.16 mW microwave power and 5 G modulation amplitude.

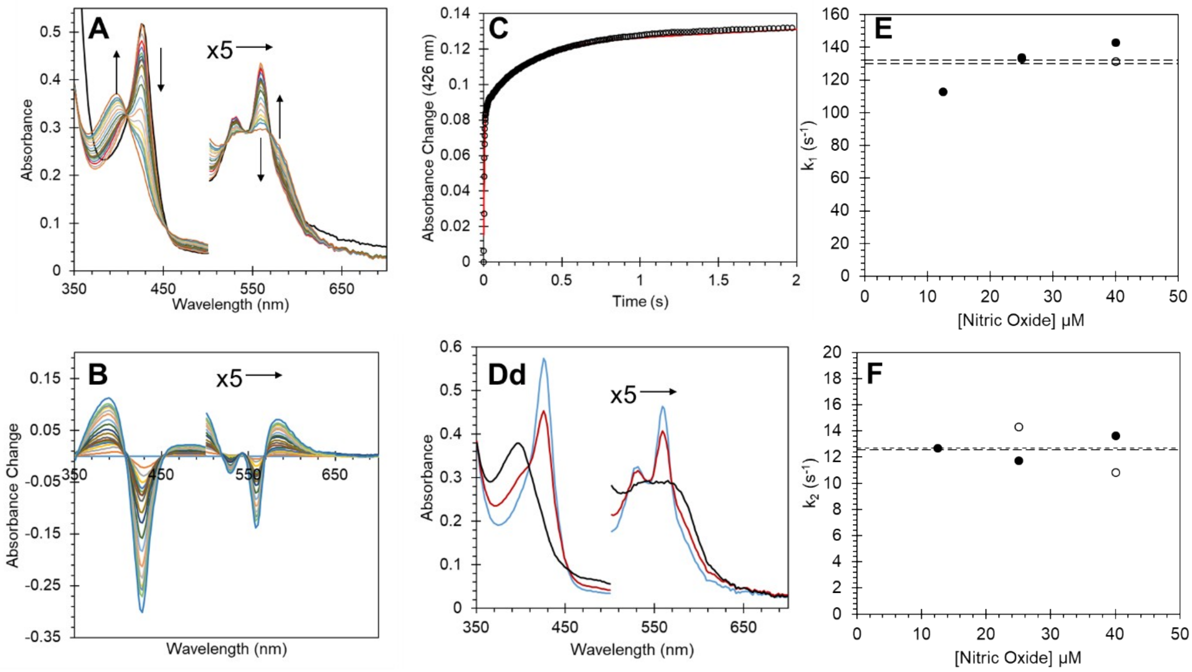

Figure S8. Binding of nitric oxide to Adgb heme iron. (A), Optical spectra of deoxyferrous Adgb-GD (5 μM) with NO (100 μM), black line represents initial deoxyferrous spectrum. (B), Difference spectra with initial ferrous protein set to zero. (C), Time course of optical changes (black circle) fitted to a double exponential function plus a linear drift (red line). (D), Global fit of initial deoxyferrous protein (blue), intermediate (red) and final ferrous-NO bound spectrum (black). The observed rate constants of the fast (E) and slow (F) phase on the binding of NO in the presence (℘) or absence (by reduction using TCEP, (☊) of disulfide bond. Dashed lines represent average observed rate constants.

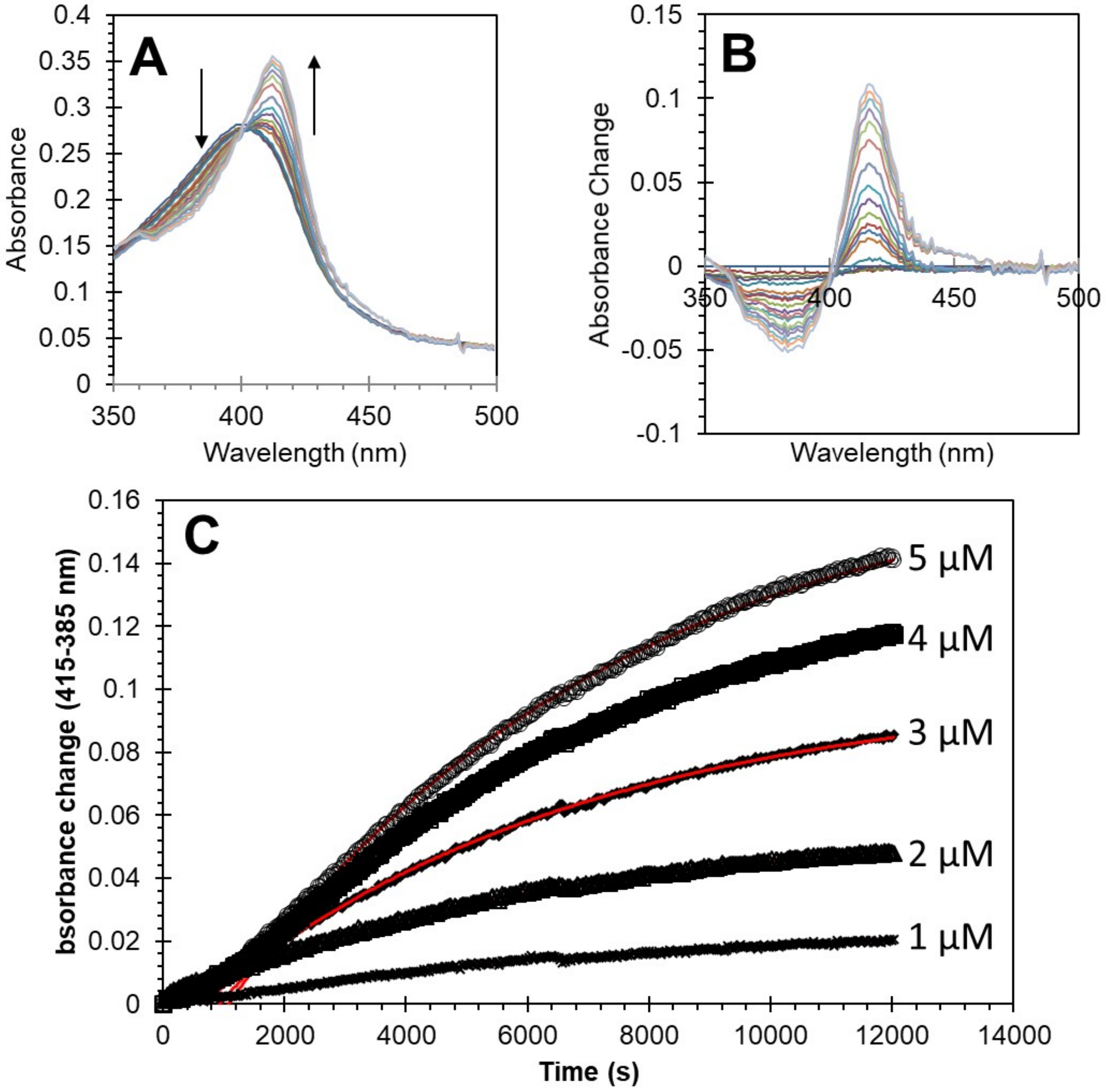

Figure S9. Oxygen ligand exchange with nitric oxide. Ferrous Adgb-GD (150 µM) in the presence of slight excess dithionite was bound to NO using 200 µM of NO from 110 µM proli-NONOate in 100 mM sodium phosphate pH 7.4, 25°C. (A) Optical changes observed following dilution of ferrous NO bound Adgb to 5 µM in oxygenated buffer. Arrows represent direction of absorbance change. (B) Difference spectra of (A). (C) Kinetic trace from (A) using 415 and 385 nm with dilutions of protein between 5 and 1 µM. Black dots are data points and red line represents fit to a single exponential function following excess NO depletion (>2000 s). Data fitted to a rate constant of 1.60 x10^{-4} s^{-1} ± 0.07 x10^{-4} s^{-1}.

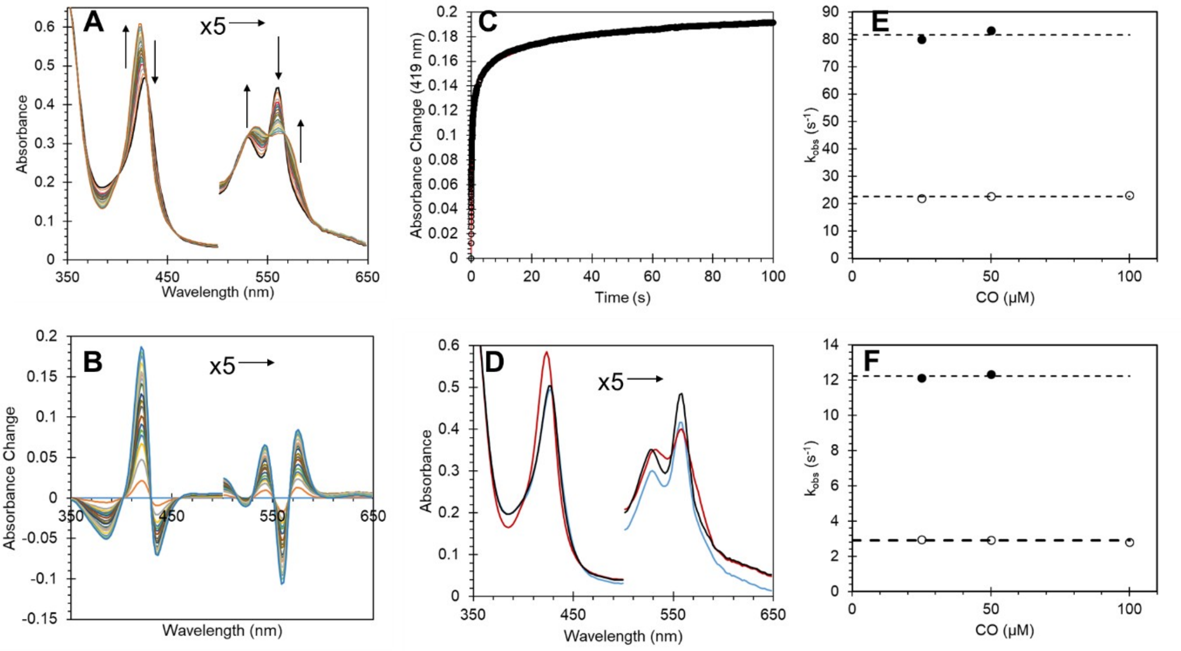

Figure S10. Binding of carbon monoxide to Adgb heme iron. (A), Optical spectra of deoxyferrous Adgb-GD (5 µM) with CO (100 µM). (B), Difference spectra with initial deoxyferrous protein set to zero. (C), Time course of optical changes (black circle) fitted to a double exponential function plus a linear drift (red line). (D), Global fit of initial deoxyferrous protein (blue), intermediate (red) and final ferrous-CO bound spectrum (black). The observed rate constants of the fast (E) and slow (F) phase on the binding of NO in the presence (☙) or absence (by reduction using TCEP, (❧) of disulfide bond. Dashed lines represent average observed rate constants.

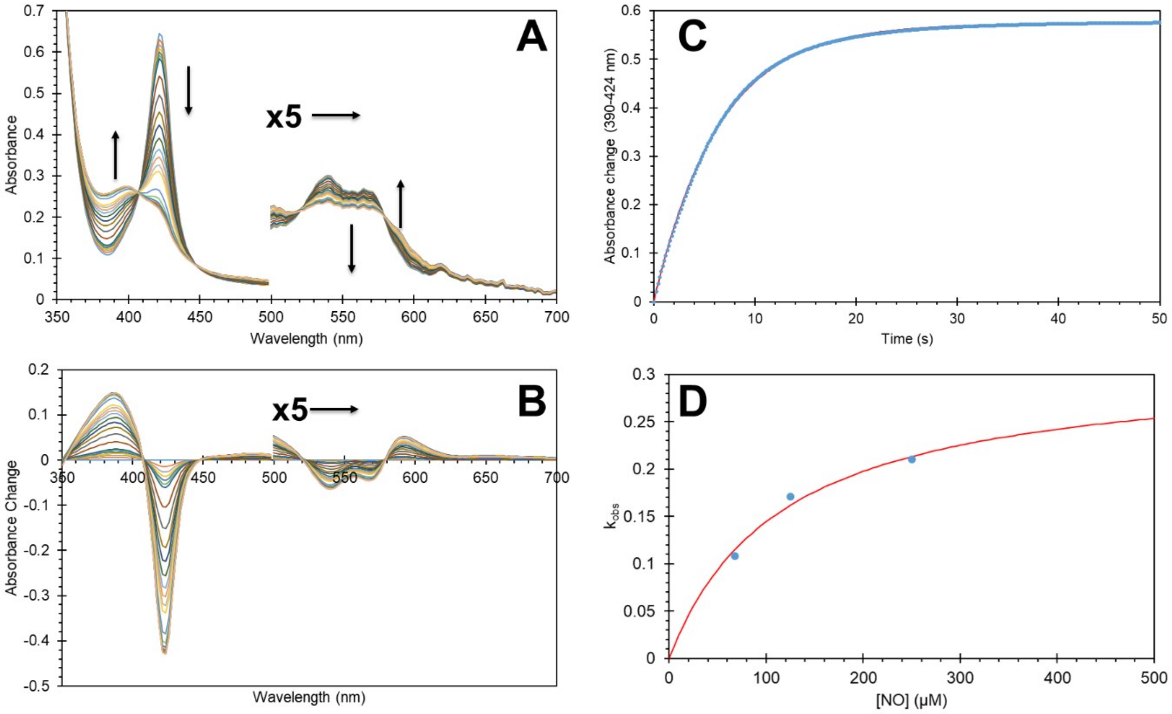

Figure S11. Nitric oxide ligand exchange with carbon monoxide. Ferrous Adgb-GD (5 μM) was saturated with CO (~1 mM) in 100 mM sodium phosphate pH 7.4, 25°C. Nitric oxide (67.5-250 μM final concentration) was added rapidly via stopped flow and the optical changes monitored. (A) Optical changes observed following addition of 250 μM NO to carbonmonoxy bound Adgb-GD. Arrows represent direction of absorbance change. (B) Difference spectra of (A). (C) Kinetic trace from (A) using 390 nm and 424 nm. Blue dots are data points and red line represents fit to a single exponential function. (D) Observed rate of NO-CO exchange as a function of NO concentration, giving a calculated CO k_{off} of 0.31 s^{-1} ± 0.05 s^{-1}

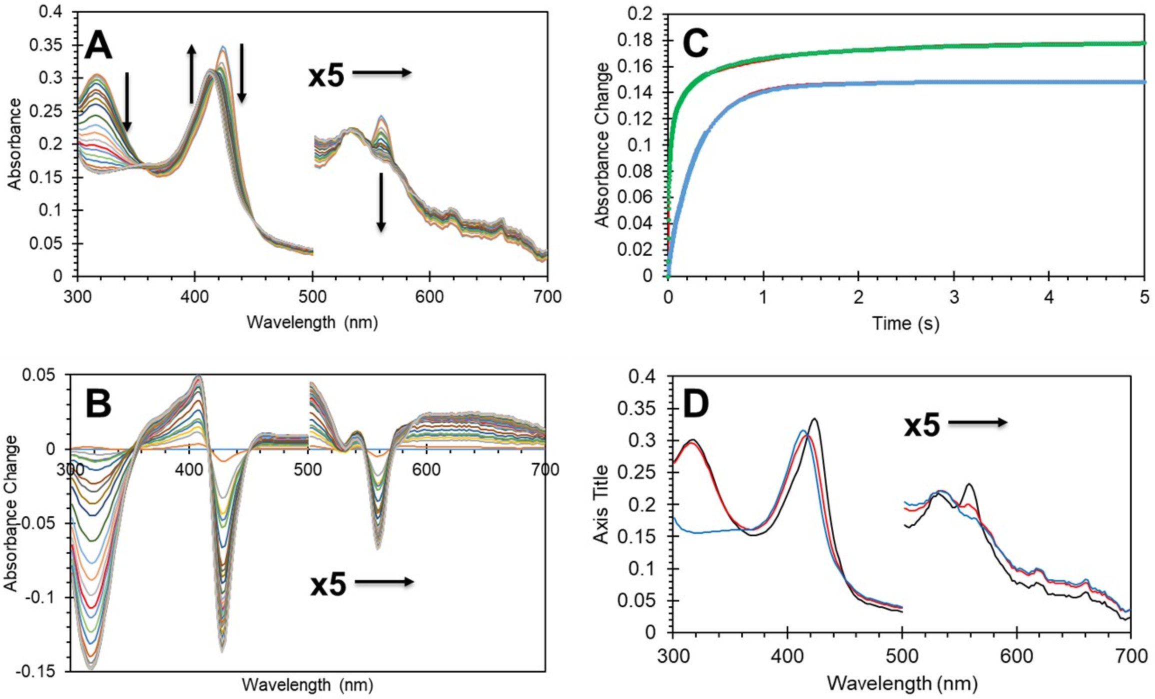

Figure S12. Rapid autoxidation of Adgb-GD by oxygen. Ferrous Adgb-GD (5 µM final) reduced with slight excess dithionite and rapidly mixed with oxygenated buffer (100 mM sodium phosphate pH 7.4, 25°C) containing 250U catalase (A). (B) Difference spectra of (A). (C) Kinetic changes in the Soret region (410-428 nm, green) and UV region (355-318 nm, blue), following heme optical changes and dithionite oxidation respectively. Red lines represent fit to double and single exponential for Soret and UV traces respectively. (D) Global analysis fit to an A→B→C sequential mechanism (black spectra is deoxyferrous Adgb-GD, red is oxyferrous and blue is ferric).

S13. References

1. A. Lock, R. Forfar, C. Weston, L. Bowsher, G. J. Upton, C. A. Reynolds, G. Ladds and A. M. Dixon, One motif to bind them: A small-XXX-small motif affects transmembrane domain 1 oligomerization, function, localization, and cross-talk between two yeast GPCRs, *Biochim Biophys Acta*, 2014, **1838**, 3036-3051.
2. S. F. Altschul, W. Gish, W. Miller, E. W. Myers and D. J. Lipman, Basic local alignment search tool, *J Mol Biol*, 1990, **215**, 403-410.
3. F. Sievers, A. Wilm, D. Dineen, T. J. Gibson, K. Karplus, W. Li, R. Lopez, H. McWilliam, M. Remmert, J. Soding, J. D. Thompson and D. G. Higgins, Fast, scalable generation of high-quality protein multiple sequence alignments using Clustal Omega, *Mol Syst Biol*, 2011, **7**, 539.
4. M. Clamp, J. Cuff, S. M. Searle and G. J. Barton, The Jalview Java alignment editor, *Bioinformatics*, 2004, **20**, 426-427.
5. N. Eswar, B. Webb, M. A. Marti-Renom, M. S. Madhusudhan, D. Eramian, M. Y. Shen, U. Pieper and A. Sali, Comparative protein structure modeling using MODELLER, *Curr Protoc Protein Sci*, 2007, **Chapter 2**, Unit 2 9.
6. B. Taddese, G. J. Upton, G. R. Bailey, S. R. Jordan, N. Y. Abdulla, P. J. Reeves and C. A. Reynolds, Do plants contain g protein-coupled receptors?, *Plant Physiol*, 2014, **164**, 287-307.
7. H. A. Watkins, M. Chakravarthy, R. S. Abhayawardana, J. J. Gingell, M. Garelja, M. Pardamwar, J. M. McElhinney, A. Lathbridge, A. Constantine, P. W. Harris, T. Y. Yuen, M. A. Brimble, J. Barwell, D. R. Poyner, M. J. Woolley, A. C. Conner, A. A. Pioszak, C. A. Reynolds and D. L. Hay, Receptor Activity-modifying Proteins 2 and 3 Generate Adrenomedullin Receptor Subtypes with Distinct Molecular Properties, *J Biol Chem*, 2016, **291**, 11657-11675.
8. B. Webb and A. Sali, Comparative Protein Structure Modeling Using MODELLER, *Curr Protoc Protein Sci*, 2016, **86**, 2 9 1-2 9 37.
9. D. W. Buchan, F. Minneci, T. C. Nugent, K. Bryson and D. T. Jones, Scalable web services for the PSIPRED Protein Analysis Workbench, *Nucleic Acids Res*, 2013, **41**, W349-357.
10. C. Cole, J. D. Barber and G. J. Barton, The Jpred 3 secondary structure prediction server, *Nucleic Acids Res*, 2008, **36**, W197-201.
11. H. Mirzaei, S. Zarbafian, E. Villar, S. Mottarella, D. Beglov, S. Vajda, I. Paschalidis, P. Vakili and D. Kozakov, Energy Minimization on Manifolds for Docking Flexible Molecules, *J Chem Theory Comput*, 2015, **11**, 1063-1076.
12. J. Jumper, R. Evans, A. Pritzel, T. Green, M. Figurnov, O. Ronneberger, K. Tunyasuvunakool, R. Bates, A. Zidek, A. Potapenko, A. Bridgland, C. Meyer, S. A. A. Kohl, A. J. Ballard, A. Cowie, B. Romera-Paredes, S. Nikolov, R. Jain, J. Adler, T. Back, S. Petersen, D. Reiman, E. Clancy, M. Zielinski, M. Steinegger, M. Pacholska, T. Berghammer, S. Bodenstein, D. Silver, O. Vinyals, A. W. Senior, K. Kavukcuoglu, P. Kohli and D. Hassabis, Highly accurate protein structure prediction with AlphaFold, *Nature*, 2021, **596**, 583-589.
13. K. Tunyasuvunakool, J. Adler, Z. Wu, T. Green, M. Zielinski, A. Zidek, A. Bridgland, A. Cowie, C. Meyer, A. Laydon, S. Velankar, G. J. Kleywegt, A. Bateman, R. Evans, A. Pritzel, M. Figurnov, O. Ronneberger, R. Bates, S. A. A. Kohl, A. Potapenko, A. J. Ballard, B. Romera-Paredes, S. Nikolov, R. Jain, E. Clancy, D. Reiman, S. Petersen, A. W. Senior, K. Kavukcuoglu, E. Birney, P. Kohli, J. Jumper and D. Hassabis, Highly accurate protein structure prediction for the human proteome, *Nature*, 2021, **596**, 590-596.
14. J. Huang and A. D. MacKerell, Jr., CHARMM36 all-atom additive protein force field: validation based on comparison to NMR data, *J Comput Chem*, 2013, **34**, 2135-2145.
15. S. Doerr, M. J. Harvey, F. Noe and G. De Fabritiis, HTMD: High-Throughput Molecular Dynamics for Molecular Discovery, *J Chem Theory Comput*, 2016, **12**, 1845-1852.

16. T. J. Dolinsky, J. E. Nielsen, J. A. McCammon and N. A. Baker, PDB2PQR: an automated pipeline for the setup of Poisson-Boltzmann electrostatics calculations, *Nucleic Acids Res*, 2004, **32**, W665-667.
17. M. H. Olsson, C. R. Sondergaard, M. Rostkowski and J. H. Jensen, PROPKA3: Consistent Treatment of Internal and Surface Residues in Empirical pKa Predictions, *J Chem Theory Comput*, 2011, **7**, 525-537.
18. W. L. Jorgensen, J. Chandrasekhar, J. D. Madura, R. W. Impey and M. L. Klein, Comparison of Simple Potential Functions for Simulating Liquid Water, *J Chem Phys*, 1983, **79**.
19. M. J. Harvey, G. Giupponi and G. D. Fabritiis, ACEMD: Accelerating Biomolecular Dynamics in the Microsecond Time Scale, *J Chem Theory Comput*, 2009, **5**, 1632-1639.
20. H. J. C. Berendsen, J. P. M. Postma, W. F. Vangunsteren, A. Dinola and J. R. Haak, Molecular-Dynamics with Coupling to an External Bath, *J Chem Phys*, 1984, **81**, 3684-3690.
21. R. J. Loncharich, B. R. Brooks and R. W. Pastor, Langevin dynamics of peptides: the frictional dependence of isomerization rates of N-acetylalanyl-N'-methylamide, *Biopolymers*, 1992, **32**, 523-535.
22. V. Krautler, W. F. Van Gunsteren and P. H. Hunenberger, A fast SHAKE: Algorithm to solve distance constraint equations for small molecules in molecular dynamics simulations, *J Comput Chem*, 2001, **22**, 501-508.
23. U. Essmann, L. Perera, M. L. Berkowitz, T. Darden, H. Lee and L. G. Pedersen, A Smooth Particle Mesh Ewald Method, *Journal of Chemical Physics*, 1995, **103**, 8577-8593.
24. W. Humphrey, A. Dalke and K. Schulten, VMD: Visual molecular dynamics, *J Mol Graph Model*, 1996, **14**, 33-38.
25. R. T. McGibbon, K. A. Beauchamp, M. P. Harrigan, C. Klein, J. M. Swails, C. X. Hernandez, C. R. Schwantes, L. P. Wang, T. J. Lane and V. S. Pande, MDTraj: A Modern Open Library for the Analysis of Molecular Dynamics Trajectories, *Biophys J*, 2015, **109**, 1528-1532.
26. E. Antonini and M. Brunori, in *Frontiers in Biology*, eds. A. Neuberger and E. L. Tatum, North-Holland publishing company, Amsterdam-London, 1971, vol. 21, pp. 13-52.
27. P. Beckerson, M. T. Wilson, D. A. Svistunenko and B. J. Reeder, Cytoglobin ligand binding regulated by changing haem-co-ordination in response to intramolecular disulfide bond formation and lipid interaction, *Biochem J*, 2015, **465**, 127-137.
28. B. J. Reeder, D. A. Svistunenko, M. A. Sharpe and M. T. Wilson, Characteristics and mechanism of formation of peroxide-induced heme to protein cross-linking in myoglobin, *Biochemistry*, 2002, **41**, 367-375.
29. C. K. Riener, G. Kada and H. J. Gruber, Quick measurement of protein sulfhydryls with Ellman's reagent and with 4,4'-dithiodipyridine, *Anal Bioanal Chem*, 2002, **373**, 266-276.
30. B. J. Reeder, D. A. Svistunenko and M. T. Wilson, Lipid binding to cytoglobin leads to a change in haem co-ordination: a role for cytoglobin in lipid signalling of oxidative stress, *Biochem J*, 2011, **434**, 483-492.
31. M. Simons, S. Gretton, G. G. A. Silkstone, B. S. Rajagopal, V. Allen-Baume, N. Syrett, T. Shaik, N. Leiva-Eriksson, L. Ronda, A. Mozzarelli, M. B. Strader, A. I. Alayash, B. J. Reeder and C. E. Cooper, Comparison of the oxidative reactivity of recombinant fetal and adult human hemoglobin: implications for the design of hemoglobin-based oxygen carriers, *Biosci Rep*, 2018, **38**.
32. L. Lobato, L. Bouzhir-Sima, T. Yamashita, M. T. Wilson, M. H. Vos and U. Liebl, Dynamics of the heme-binding bacterial gas-sensing dissimilative nitrate respiration regulator (DNR) and activation barriers for ligand binding and escape, *J Biol Chem*, 2014, **289**, 26514-26524.
33. S. Nicolis, E. Monzani, C. Ciaccio, P. Ascenzi, L. Moens and L. Casella, Reactivity and endogenous modification by nitrite and hydrogen peroxide: does human neuroglobin act only as a scavenger?, *Biochem J*, 2007, **407**, 89-99.

34. A. Fago, C. Hundahl, S. Dewilde, K. Gilany, L. Moens and R. E. Weber, Allosteric regulation and temperature dependence of oxygen binding in human neuroglobin and cytoglobin. Molecular mechanisms and physiological significance, *J Biol Chem*, 2004, **279**, 44417-44426.
35. D. Hamdane, L. Kiger, S. Dewilde, B. N. Green, A. Pesce, J. Uzan, T. Burmester, T. Hankeln, M. Bolognesi, L. Moens and M. C. Marden, The redox state of the cell regulates the ligand binding affinity of human neuroglobin and cytoglobin, *J Biol Chem*, 2003, **278**, 51713-51721.
36. S. Herold, A. Fago, R. E. Weber, S. Dewilde and L. Moens, Reactivity studies of the Fe(III) and Fe(II)NO forms of human neuroglobin reveal a potential role against oxidative stress, *J Biol Chem*, 2004, **279**, 22841-22847.
37. D. J. Baker and P. J. Lindop, Oxygen cathode measurements in the mouse testis, *Phys Med Biol*, 1970, **15**, 263-270.
38. B. J. Reeder and J. Ukeri, Strong modulation of nitrite reductase activity of cytoglobin by disulfide bond oxidation: Implications for nitric oxide homeostasis, *Nitric Oxide*, 2018, **72**, 16-23.
39. P. Corti, M. Ieraci and J. Tejero, Characterization of zebrafish neuroglobin and cytoglobins 1 and 2: Zebrafish cytoglobins provide insights into the transition from six-coordinate to five-coordinate globins, *Nitric Oxide*, 2016, **53**, 22-34.
40. P. Corti, J. Xue, J. Tejero, N. Wajih, M. Sun, D. B. Stolz, M. Tsang, D. B. Kim-Shapiro and M. T. Gladwin, Globin X is a six-coordinate globin that reduces nitrite to nitric oxide in fish red blood cells, *Proc Natl Acad Sci U S A*, 2016, **113**, 8538-8543.
41. Z. Huang, S. Shiva, D. B. Kim-Shapiro, R. P. Patel, L. A. Ringwood, C. E. Irby, K. T. Huang, C. Ho, N. Hogg, A. N. Schechter and M. T. Gladwin, Enzymatic function of hemoglobin as a nitrite reductase that produces NO under allosteric control, *J Clin Invest*, 2005, **115**, 2099-2107.
42. M. Tiso, J. Tejero, S. Basu, I. Azarov, X. Wang, V. Simplaceanu, S. Frizzell, T. Jayaraman, L. Geary, C. Shapiro, C. Ho, S. Shiva, D. B. Kim-Shapiro and M. T. Gladwin, Human neuroglobin functions as a redox-regulated nitrite reductase, *J Biol Chem*, 2011, **286**, 18277-18289.
43. J. W. Petrich, J. C. Lambry, S. Balasubramanian, D. G. Lambright, S. G. Boxer and J. L. Martin, Ultrafast measurements of geminate recombination of NO with site-specific mutants of human myoglobin, *J Mol Biol*, 1994, **238**, 437-444.
44. M. Negrerie, V. Berka, M. H. Vos, U. Liebl, J. C. Lambry, A. L. Tsai and J. L. Martin, Geminate recombination of nitric oxide to endothelial nitric-oxide synthase and mechanistic implications, *J Biol Chem*, 1999, **274**, 24694-24702.
45. M. H. Vos, Ultrafast dynamics of ligands within heme proteins, *Biochim Biophys Acta*, 2008, **1777**, 15-31.
46. M. Negrerie, L. Bouzhir, J. L. Martin and U. Liebl, Control of nitric oxide dynamics by guanylate cyclase in its activated state, *J Biol Chem*, 2001, **276**, 46815-46821.
47. S. G. Kruglik, J. C. Lambry, S. Cianetti, J. L. Martin, R. R. Eady, C. R. Andrew and M. Negrerie, Molecular basis for nitric oxide dynamics and affinity with Alcaligenes xylosoxidans cytochrome c, *J Biol Chem*, 2007, **282**, 5053-5062.
48. D. Hoogewijs, B. Ebner, F. Germani, F. G. Hoffmann, A. Fabrizius, L. Moens, T. Burmester, S. Dewilde, J. F. Storz, S. N. Vinogradov and T. Hankeln, Androglobin: a chimeric globin in metazoans that is preferentially expressed in Mammalian testes, *Mol Biol Evol*, 2012, **29**, 1105-1114.
